# Prognostic relevance of miRNA-155 methylation in anaplastic glioma

**DOI:** 10.18632/oncotarget.13452

**Published:** 2016-11-18

**Authors:** Maximilian Georg Schliesser, Rainer Claus, Thomas Hielscher, Christiane Grimm, Dieter Weichenhan, Jonas Blaes, Benedikt Wiestler, Peter Hau, Johannes Schramm, Felix Sahm, Elisa K. Weiß, Markus Weiler, Constance Baer, Friederike Schmidt-Graf, Gabriele Schackert, Manfred Westphal, Anne Hertenstein, Patrick Roth, Norbert Galldiks, Christian Hartmann, Torsten Pietsch, Joerg Felsberg, Guido Reifenberger, Michael Christoph Sabel, Frank Winkler, Andreas von Deimling, Christoph Meisner, Peter Vajkoczy, Michael Platten, Michael Weller, Christoph Plass, Wolfgang Wick

**Affiliations:** ^1^ Department of Neurology, Heidelberg University Hospital and German Cancer Consortium, Clinical Cooperation Units, Germany; ^2^ Department of Neuropathology, Heidelberg University Hospital and German Cancer Consortium, Clinical Cooperation Units, Germany; ^3^ Clinical Cooperation Unit of Neurooncology, German Cancer Research Center (DKFZ), Heidelberg, Germany; ^4^ Clinical Cooperation Unit of Neuropathology, German Cancer Research Center (DKFZ), Heidelberg, Germany; ^5^ Clinical Cooperation Unit of Neuroimmunology and Brain Tumor Immunology, German Cancer Research Center (DKFZ), Heidelberg, Germany; ^6^ Division of Epigenomics and Cancer Risk Factors, German Cancer Research Center (DKFZ), Heidelberg, Germany; ^7^ Division of Biostatistics, German Cancer Research Center (DKFZ), Heidelberg, Germany; ^8^ Department of General Neurology, University Hospital Tübingen, Germany; ^9^ Department of Biostatistics, University Hospital Tübingen, Germany; ^10^ Neurology Clinic, Regensburg University, Regensburg, Germany; ^11^ Neurosurgery Clinic, University of Bonn Medical Center, TU Munich, Munich, Germany; ^12^ Neurology Clinic, TU Munich, Munich, Germany; ^13^ Neurosurgery Clinic, Dresden University Medical Center, Germany; ^14^ Neurosurgery Clinic, University Clinic Hamburg, Eppendorf, Germany; ^15^ Department of Neurology, University Hospital Zurich, Zurich, Switzerland; ^16^ Neurology Clinic, Cologne University, Cologne, Germany; ^17^ Department for Neuropathology, Institute of Pathology, Medical University of Hannover, Hannover, Germany; ^19^ Department of Neuropathology, University of Bonn Medical Center, Bonn, Germany; ^20^ Department of Neuropathology, Heinrich-Heine-University, Germany; ^21^ Department of Neurosurgery, Heinrich-Heine-University, Germany; ^22^ DKTK, Partner Site Essen/Düsseldorf, Düsseldorf, Germany; ^23^ Neurosurgery Clinic, Charité, Berlin, Germany; ^24^ Department Hematology, Oncology and Stem Cell Transplantation, University Hospital Freiburg, Germany; ^25^ Department of Diagnostic and Interventional Neuroradiology, Klinikum rechts der Isar der Technischen Universität München, Munich, Germany

**Keywords:** anaplastic glioma, miR-155, IDH, NFκB, NOA-04

## Abstract

The outcome of patients with anaplastic gliomas varies considerably depending on single molecular markers, such as mutations of the *isocitrate dehydrogenase* (*IDH*) genes, as well as molecular classifications based on epigenetic or genetic profiles. Remarkably, 98% of the RNA within a cell is not translated into proteins. Of those, especially microRNAs (miRNAs) have been shown not only to have a major influence on physiologic processes but also to be deregulated and prognostic in malignancies.

To find novel survival markers and treatment options we performed unbiased DNA methylation screens that revealed 12 putative miRNA promoter regions with differential DNA methylation in anaplastic gliomas. Methylation of these candidate regions was validated in different independent patient cohorts revealing a set of miRNA promoter regions with prognostic relevance across data sets. Of those, miR-155 promoter methylation and miR-155 expression were negatively correlated and especially the methylation showed superior correlation with patient survival compared to established biomarkers.

Functional examinations in malignant glioma cells further cemented the relevance of miR-155 for tumor cell viability with transient and stable modifications indicating an onco-miRNA activity. MiR-155 also conferred resistance towards alkylating temozolomide and radiotherapy as consequence of nuclear factor (NF)κB activation.

Preconditioning glioma cells with an NFκB inhibitor reduced therapy resistance of miR-155 overexpressing cells. These cells resembled tumors with a low methylation of the miR-155 promoter and thus mir-155 or NFκB inhibition may provide treatment options with a special focus on patients with *IDH* wild type tumors.

## INTRODUCTION

Anaplastic gliomas of World Health Organization (WHO) grade III are currently subdivided into anaplastic astrocytic and oligodendroglial (and mixed) tumors [1]. The considerable interobserver variation for grading and typing of gliomas [2], the variability of outcomes within the subgroups and a paucity of therapeutically attractive targets derived from this framework have triggered studies suggesting a molecularly-based classification for grade II and III gliomas. This histological classification and the underlying mutations in the *isocitrate dehydrogenase* (IDH) genes 1 and 2 resulting in a glioma CpG island methylator phenotype (GCIMP) [3], 1p/19q co-deletions, as well as mutually exclusive mutations in *telomerase reverse transcriptase* (*TERT*) and *alpha-thalassemia/mental retardation syndrome X-linked* (*ATRX*) genes [4, 5] are providing a clinically useful prognostic framework [6]. Indeed, gliomas across histological subtypes with an *IDH* mutation carry a very similar epigenetic profile [7]. This and other studies [8] suggest that *IDH* mutant gliomas form a biologically distinct entity. However, there is still value to the WHO grading [52] and a need for deeper understanding of the molecular biology of gliomas outside the known genetic, epigenetic and transcriptional changes. Noteworthy, the majority of transcripts within a cell does not represent protein-coding but actually non-coding RNA (ncRNA) [9–11]. Amongst those, miRNA gained major attraction as they act as mainly inhibitory modifiers of translation by steric hindrance of the ribosome or by prompting the mRNA degradation [12, 13] which are of paramount relevance in cancer [14]. In a tumor setting the phenotypic effect of miRNAs depends on the function of the inhibited mRNAs.

The aim of the present work was to better understand the transcriptional regulation of miRNAs in gliomas, find novel survival marker as well as treatment options.

## RESULTS

### Differential methylation of miRNAs in anaplastic gliomas

**Figure 1:**
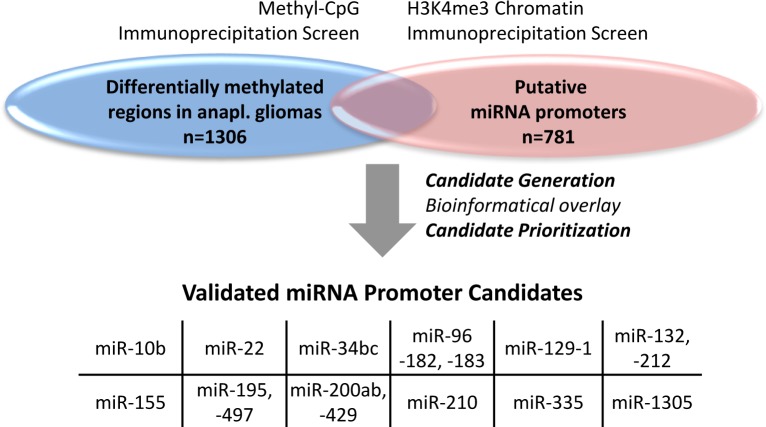
miRNA candidates were generated by the overlay of two data sets The initial list of differentially methylated miRNA promoter candidates in anaplastic (anapl.) gliomas originated from an overlay of two distinct screens: a methyl-CpG immunoprecipitation screen for differentially methylated regions in anaplastic gliomas from the NOA-04 trial (*n* = 4; healthy *n* = 1) *vs*. an H3K4me3 chromatin immunoprecipitation data set from cell lines of different origin (*n* = 6) and chronic lymphocytic leukemia patients (*n* = 24; healthy *n* = 10) for putative miRNA promoters. From the 29 candidates (Supplementary Figure 1) produced by this overlay the 12 most favorable candidate regions were validated by MassARRAY.

The overlay of differentially methylated regions (DMRs) detected in tissue samples from the NOA-04 trial and putative miRNA promoter regions generated an initial list of 29 differentially methylated candidate miRNA promoter regions. The DMR data set was derived from a MCIp-based DNA methylation profiling and the promoter set had been previously identified in an H3K4me3 ChIP screen [18]. The candidates were prioritized and checked for promoter activity taking into account additional factors: a favorable distance and orientation of the miRNA gene, adjacent CpG islands, the degree of DNase hypersensitivity and vertebrate conservation of the target region. The 12 most promising miRNA-associated candidate regions with differential methylation in anaplastic gliomas were selected for in-depth analysis (Figure 1, Supplementary Figure 1) and validated by quantitative DNA methylation analysis using the MassARRAY technology.

### Prognostic relevance of miRNA methylation in anaplastic gliomas

**Figure 2:**
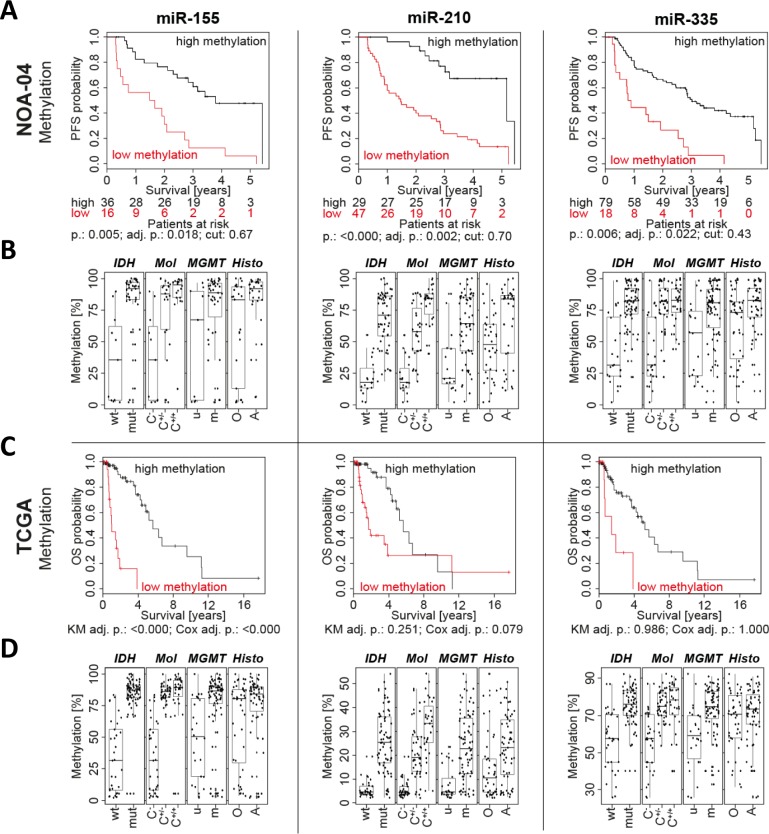
Low promoter methylation of miR-155, miR-210 and miR-335 was associated with a short patient survival Three promising miRNA candidates were selected and their Kaplan-Meier estimate from the NOA-04 patients calculated **A.** The statistically determined cutpoint (cut) depicts the threshold separating the patients in low and high methylation. The number of patients in each group is listed below each graph. Full data for all amplicons are in the supplement. Furthermore, miRNA candidate methylation was plotted according to the *IDH* mutation status, molecular classification (Mol) with CpG island methylator phenotype (CIMP) and 1p/19q codeletion status [28], *MGMT* promoter methylation and tumor histology (histo) **B.** Additionally, the miRNA methylation for miR-155, miR-210 and miR-335 was correlated with the overall survival (OS) by Kaplan-Meier analysis (KM) and Cox regression in 90 patients with anaplastic gliomas available from The Cancer Genome Atlas (TCGA) **C.** and plotted like for the NOA-04 patients. PFS: progression free survival; p.: individual p-value; adj. p.: p-value adjusted for testing of multiple amplicons, CpG fragments and cutpoints; *IDH*: *Isocitrate dehydrogenase*; mut: mutated; wt: wild type; C-: CIMP negative; C+/−: CIMP positive, non-codeleted; C+/+: CIMP positive, 1p/19q codeleted; *MGMT*: *O6-methylguanine-DNA methyltransferase*; m: methylated; u: unmethylated; O(A): oligodendroglioma and oligoastrocytoma; A: astrocytoma; r: Pearson's r correlation.

The candidate regions were first analyzed in 106 patients with anaplastic gliomas from the NOA-04 trial (Table 1, Supplementary Figure 2, Figure 2A; see Supplement for patients' characteristics). The full statistics with all analyzed CpGs and amplicons is available in Supplementary Table 1. Due to differences in the sample, amplicon and procedure quality reduced number of patients with methylation data are present for different miRNA candidates of the same patient cohort. For all DMR, except the one associated with miR-10b, a high methylation was associated with a longer progression free survival (PFS) and overall survival (OS). The prognostic relevance of selected regions was validated in an independent anaplastic glioma patient samples (*n* = 82) from the GGN [16] using the same primers and settings as for the initial NOA-04 patients. In the confirmatory analysis, low methylation levels at the miR-155 and miR-210 promoters were significantly associated with worse PFS and OS (Table 1, Supplementary Figure 3; see Supplement for patients' characteristics). Methylation levels of the miR-335 promoter region were merely correlated with OS.

**Table 1:** Prognostic miRNA promoter methylation was determined for patients with anaplastic gliomas of the NOA-04 trial and validated in a GGN cohort

NOA-04		Progression Free Survival				Overall Survival			
		Obs	HazR	95% CI	p-value adj	Obs	HazR	95% CI	p-value adj
	**miR-10b**	91	1.6	[1.05, 2.45]	**0.080**	91	1.42	[0.84, 2.39]	**0.386**
	**miR-22**	71	0.45	[0.28, 0.73]	**0.005**	71	0.32	[0.16, 0.64]	**0.005**
	**miR-34bc**	98	0.69	[0.48, 0.98]	**0.093**	98	0.82	[0.54, 1.26]	**0.616**
	**miR-96, −182, −183**	105	0.54	[0.34, 0.85]	**0.028**	105	0.5	[0.27, 0.91]	**0.074**
	**miR-129-1**	104	0.89	[0.79, 1.01]	**0.175**	104	0.95	[0.79, 1.13]	**0.702**
	**miR-132, −212**	85	0.89	[0.62, 1.29]	**0.694**	85	0.75	[0.44, 1.28]	**0.556**
	**miR-155**	52	0.36	[0.23, 0.56]	**<0.000**	52	0.39	[0.22, 0.68]	**0.005**
	**miR-195, −497**	87	0.8	[0.61, 1.06]	**0.232**	87	0.72	[0.48, 1.08]	**0.249**
	**miR-200a+b, −429**	98	0.85	[0.66, 1.09]	**0.347**	98	0.91	[0.65, 1.26]	**0.702**
	**miR-210**	76	0.23	[0.13, 0.41]	**<0.000**	76	0.21	[0.10, 0.43]	**0.001**
	**miR-335**	97	0.55	[0.40, 0.77]	**0.002**	97	0.5	[0.33, 0.74]	**0.004**
	**miR-1305**	51	0.56	[0.32, 0.95]	**0.084**	51	0.53	[0.26, 1.08]	**0.192**

GGN		Progression Free Survival				Overall Survival			
		Obs	HazR	95% CI	p-value adj	Obs	HazR	95% CI	p-value adj
	**miR-10b**	79	1.51	[1.00, 2.26]	**0.089**	79	1.7	[0.96, 3.00]	**0.105**
	**miR-34bc**	81	0.86	[0.57, 1.30]	**0.576**	81	0.84	[0.47, 1.53]	**0.639**
	**miR-96, −182, −183**	80	0.76	[0.49, 1.19]	**0.306**	80	0.78	[0.42, 1.42]	**0.497**
	**miR-129-1**	78	0.67	[0.46, 0.99]	**0.089**	78	0.48	[0.28, 0.83]	**0.018**
	**miR-155**	80	0.82	[0.70, 0.96]	**0.037**	80	0.72	[0.60, 0.87]	**0.004**
	**miR-210**	80	0.46	[0.28, 0.75]	**0.022**	80	0.27	[0.14, 0.53]	**0.002**
	**miR-335**	81	0.75	[0.54, 1.03]	**0.114**	81	0.6	[0.40, 0.90]	**0.030**

The methylation of the candidate regions was analyzed by MassARRAY in patient samples of the NOA-04 cohort [51] and afterwards validated in patients from the GGN [49]. By a Cox regression model the mean methylation of each candidate region was correlated with the progression free and overall survival and the p-value corrected for testing of multiple candidates. Significant correlations were denoted in grey (adj. p-value < 0.05). The results for all analyzed CpGs and amplicons is given in the supplement. Obs: observation, number of patients with full data; HazR: Hazard ratio; CI: confidence interval; p-value adj: p-value adjusted for testing of multiple amplicons and CpG fragments

Correlation with clinically relevant markers showed that *IDH* mutation with the CpG island methylator phenotype (CIMP), 1p/19q codeletion, *MGMT* promoter methylation and oligodendroglial histology were associated with high miRNA-associated 5′-region methylation levels (Figure 2B).

Furthermore, the relevance of miRNA promoter methylation for patients with anaplastic glioma was assessed in data available from The Cancer Genome Atlas (TCGA). The analysis on TCGA data confirmed the negative correlation for miR-155-associated methylation in WHO grade III glioma and also showed a lower but significant correlation for methylation of the miR-335 promoter region (Figure 2C). Likewise, also the correlation with clinical markers resembled the NOA-04 situation (Figure 2D).

In order to test a differential impact on radio- or chemotherapy, samples from NOA-04 patients were split according to the first line treatment, which showed that the survival advantage of the candidate methylation sites was independent of treatment modality (first line chemo- or radiotherapy) (Supplementary Figure 4). To assess an age- or WHO grade-related effect, candidate regions were tested on 101 patient samples from the NOA-8 trial for patients > 65 years of age with mainly glioblastoma (89 %) or anaplastic astrocytoma (11 %) [15]. In these patients no correlation of the miRNA methylation status with survival was seen (Supplementary Table 2).

Moreover, testing the prognostic relevance of the miR-155-, miR-210- and miR-335- associated 5′ region DNA methylation in glioblastoma data available from TCGA did not produce significant results for WHO grade IV patients (Supplementary Table 3).

### Correlation of miRNA-associated DNA methylation and miRNA expression

**Figure 3:**
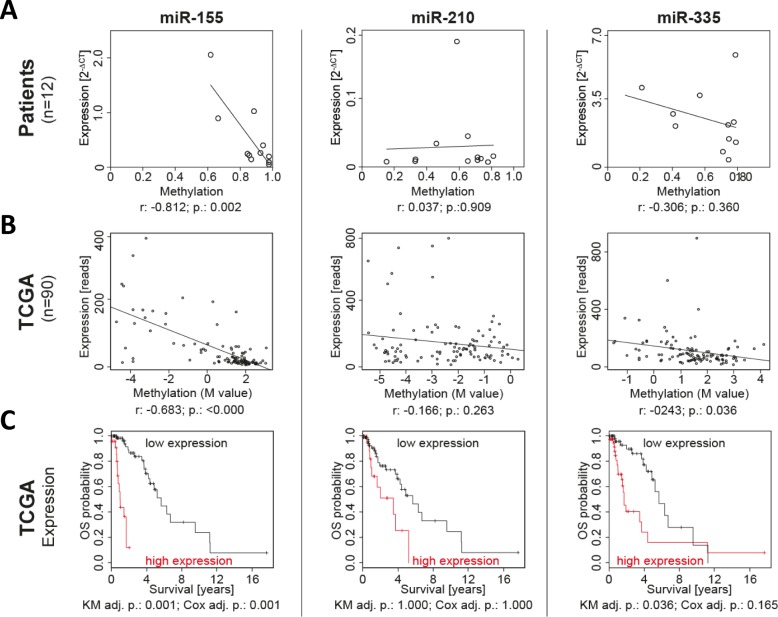
miR-155 promoter methylation and expression were negatively correlated and prognostic in patients In a set of 12 fresh frozen anaplastic glioma samples miRNA expression was determined by qPCR and methylation by MassARRAY **A.** Additionally, the promoter methylation and miRNA expression was correlated in the 90 TCGA patients with anaplastic gliomas **B.** Moreover, the prognostic relevance of the miRNA expression for overall survival (OS) was calculated by Kaplan-Meier analysis (KM) and Cox regression in the 90 patients from TCGA **C.** r: Pearson's r correlation; p.: p-value; adj. p.: p-value adjusted for testing of multiple variables.

Having established and validated the prognostic relevance of differential miR-155, miR-210 and miR-335 promoter region methylation, we aimed at deciphering the functional impact of DNA methylation differences. MiRNA promoter methylation and miRNA expression were assessed in 12 fresh frozen glioma samples (Figure 3A). For all candidates the major mature form of the respective miRNA transcript was analyzed and if not otherwise specified, the miRNA name refers to this form throughout the paper (miR-155 for miR-155-5p, miR-210 for miR-210-3p and miR-335 for miR-335-5p).

High methylation at the miR-155 promoter region was linked to low miRNA expression. In addition, the correlation of methylation and expression was performed with anaplastic glioma patient data available from TCGA, which verified the negative correlation for miR-155 and also showed a weaker but significant correlation for miR-335 (Figure 3B). Moreover, TCGA data showed that miR-155 expression is prognostic and that anaplastic glioma patients with a high miR-155 expression feature a poor survival (Figure 3C).

Relevance of promoter methylation for miRNA expression was also shown in cell lines. Decitabine (5-aza-2'-deoxycytidine) was applied and DNA methylation was determined by MassARRAY and miRNA expression by qPCR (Supplementary Figure 5, 6). Cells with an initially low miR-155 expression but high methylation of the miR-155 promoter region (T98G and U251MG) showed a strong increase in miR-155 expression after demethylation. For miR-335 also an increase from low to higher expression was seen after decitabine treatment of highly methylated cell lines.

### miR-155 promoter methylation and expression is superior to established prognostic markers

**Figure 4:**
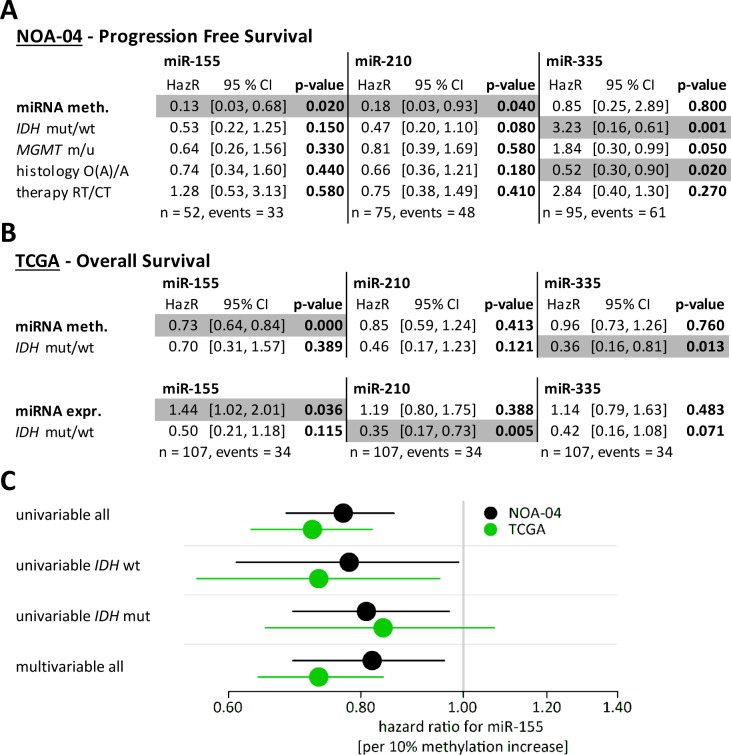
miR-155 promoter methylation and expression were strong prognostic factors even in the presence of established marker Multivariate Cox regression models were performed to test the prognostic relevance of the miRNA methylation together with established marker. Candidate methylation (meth.), *IDH* mutation status, *MGMT* promoter methylation status, histopathology and first line treatment were taken into account for the NOA-04 patients (A). From the TCGA data set the miRNA methylation and miRNA expression (expr.) were compared against the *IDH* mutation status (B). Significant correlations were denoted in grey (*p*-value < 0.05). Both multivariable analyses were additionally performed using the molecular classification with CpG island methylator phenotype (CIMP) and 1p/19q codeletion status (Supplementary Table 4) [28]. Apart from the uni- and mulitvariable Cox regression analysis and all patients, also the Hazard ratio of the miR-155 promoter methylation (per 10% methylation increase) was calculated for the *IDH* wildtype and mutant patients of the NOA-04 cohort and the TCGA data. HazR: Hazard ratio; CI: confidence interval; mut: mutated; wt: wild type; m: methylated; u: unmethylated; O(A): oligodendroglioma and oligoastrocytoma; A: astrocytoma; RT: radiotherapy; CT: chemotherapy; n: number of patients with data for all five factors; events: number of patients with progress/death.

In the next step, miRNA markers were analyzed in a multivariable analysis on PFS imputing all significant co-variables form the NOA-04 trial, namely histology, first-line therapy, *IDH* mutation and *MGMT* promoter methylation status. Putative promoter methylation of miR-155 and miR-210 were the only significant prognostic factors in the respective analyses (Figure 4A). PFS was preferred over OS for the NOA-04 calculations since the number of OS events is still too low. In addition, we analyzed the TCGA data taking *IDH* status into account (Figure 4B). Both, miR-155 promoter methylation and miR-155 expression were significant prognosticators in this calculation, while methylation and expression of miR-210 and miR-335 were not.

Additionally for both NOA-04 and TCGA patients, the miRNA methylation markers were analyzed applying the newly established molecular classification [28] (Supplementary Table 4). Also in this multivariable analysis miR-155 promoter methylation was superior.

The calculations based on the GGN patient cohort investigated here were not significant in the multivariable Cox analysis (Supplementary Table 5). However, when the prognostic relevance of *IDH* mutation status and *MGMT* promoter methylation were tested by univariable Cox analysis in this cohort, both were not significant (Supplementary Table 5). This strongly indicates a non-regular patient distribution in the investigated subset of the GGN cohort concerning these established markers. Thus, the multivariable analysis on the GGN patients is to be handled with care.

To further determine the *IDH* independent relevance of miR-155 the prognostic value of miR-155 was not only analyzed by univariable and multivariable Cox regression on all patients but also after separation into *IDH* wild type and *IDH* mutant patients (Figure 4C). In the *IDH* wild type patients of the NOA-04 and TCGA cohorts, miR-155 promoter methylation still suggests prognostic relevance. In this patient group also a highly differential miR-155 expression had been observed (Figure 2B).

### miR-155 is active as onco-miRNA

**Figure 5:**
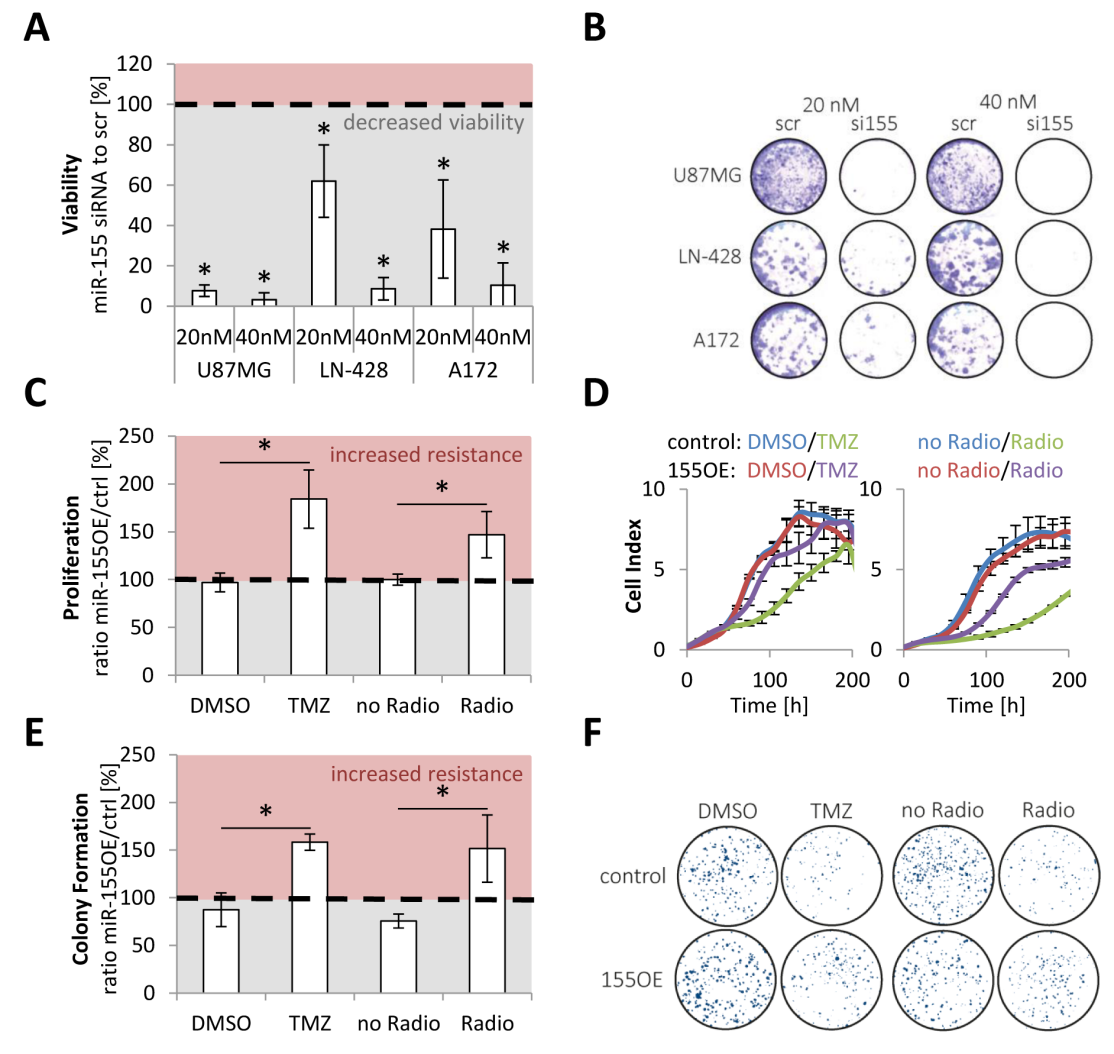
miR-155 was active as onco-miRNA and conveyed therapy resistance miR-155 high expressing glioma cell lines (U87MG, LN-428 and A172) were transfected with scrambled (scr) and anti-miR-155 siRNA (si155) and the viability of the cells measured with AlamarBlue **A.** Additionally, a representative staining is shown **B.** Stable miR-155 overexpressing (155OE) T98G were generated and the proliferation measured in comparison to control cells (ctrl) in the Real Time Cell Analyzer (RTCA) after chemo- (TMZ) and radiotherapy treatment **C.** Moreover, colony formation assays were performed with the control and 155 overexpressing T98G **E.** For both assays a representative experiment is given **D.**, **F.** *p*-values < 0.05 are marked by an asterisk. TMZ: temozolomide.

After establishing miR-155 as strong prognostic marker, even in comparison to established markers, the aim was to better understand the biology behind miR-155 and deduce its role for reduced patient survival. Glioma cell (U87MG, A172 and LN-428) viability was markedly reduced after miR-155 levels were transiently knocked down by siRNA (Figure 5A, Supplementary Figure 7).

For in-depth functional analyses a stable overexpression of miR-155 in the glioma cell line T98G was established (Supplementary Figure 8) and the proliferation and clonogenicity analyzed in the presence of temozolomide and radiotherapy (Figure 5C-5F). Proliferation and clonogenicity assays revealed that miR-155 confers resistance to both treatment modalities.

### miR-155 as master modulator induces NFκB activity

**Figure 6:**
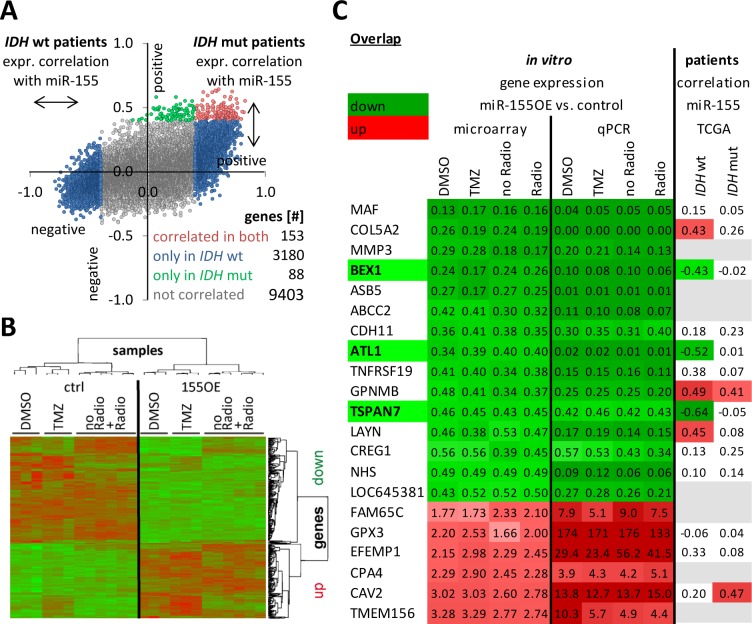
Genes were consistently deregulated according to miR-155 expression status in patients and *in vitro* Gene expression data from patients with anaplastic gliomas of TCGA was used to calculate the degree of positive (0.4 to 1) or negative (−1 to −0.4) correlation with miR-155 expression **A.** The analysis was performed individually for *IDH* mutant (mut) and *IDH* wild type (wt) patients. Each dot represents a gene and displays its correlation with miR-155 expression in *IDH* mutant (y-axis) and wild type patients (x-axis). Microarray analyses were performed with control (ctrl) T98G and miR-155 overexpressing (OE) T98G after treatment with chemo- (TMZ) or radiotherapy including the respective mock treatments **B.** The transcriptome of the samples from three independent experiments were clustered in a heatmap. Next, the *in vitro* microarray and the correlation in patients were combined to uncover relevant transcriptional changes due to miR-155 expression **C.** Candidates were validated by qPCR. The microarray and qPCR data represent fold change of the gene expression from the miR-155 overexpressing cells to the control cells. For the patient data the degree of correlation of the expression of each gene with the miR-155 expression is given. Marked are BEX1, ATL1 and TSPAN7 with a significant miR-155 dependent down deregulation in all *in vitro* treatment groups and the *IDH* wild type patients. An upregulated expression or positive correlation (cor.) with miR-155 expression was colored in red and the opposite in green. Genes without data were denoted in gray. TMZ: temozolomide; mut: mutation.

To further elucidate the effect of miR-155 overexpression in glioma cells the transcriptional changes upon miR-155 expression were analyzed *in vitro* and in patient-derived tissue data. To capture the latter, the anaplastic glioma TCGA data was used to determine which genes were positively or negatively expressed together with miR-155. Since recent data of our group [29] and others [30–32] indicate that the *IDH* or GCIMP status separates two distinct patient groups, tissues were split into *IDH* wild type and *IDH* mutant tumors. Especially in the *IDH* wild type patients a high number of genes were found whose expression was either positively (0.4 to 1) or negatively (−1 to −0.4) correlated to the expression of miR-155 (Figure 6A). This patient group had initially also featured a differential miR-155 promoter methylation in contrast to the *IDH* mutant tumors, which had a uniformly high miR-155 promoter methylation (Figure 2B).

For the *in vitro* situation microarray expression analyses were performed with the control and miR-155 overexpressing cells after mock treatment and chemo- or radiotherapy showing a high number of transcriptional changes upon miR-155 expression in the heatmap (Figure 6B).

**Figure 7:**
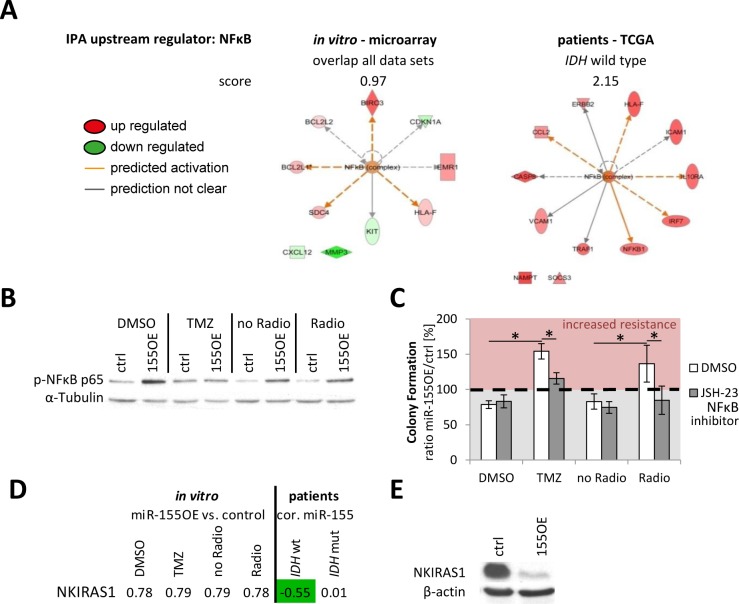
NFkB activation was found as common theme upon miR-155 expression The *in vitro* and patient data were analyzed with Ingenuity Pathway Analysis (IPA). From the microarray genes consistently deregulated in all groups were used (before multiple testing correction). The analysis revealed in both sets as upstream regulator the activation of NFκB upon miR-155 expression **A.** Immunoblot analysis of the p65 subunit phosphorylation (p-NFκB p65) confirmed the NFκB activation in miR-155 overexpressing (155OE) cells in comparison to control (ctrl) cells **B.** The cells were seeded for colony formation assay and pretreated with the NFκB inhibitor JSH-23 or DMSO as control followed by the standard chemo- (TMZ) and radiotherapy **C.** The ratio of colonies from miR-155 overexpressing cells (OE) to control cells (ctrl) was calculated for each treatment. *p*-values < 0.05 are marked by an asterisk. NKIRAS1 was recently confirmed as miR-155 target [33]. Apart from analysis on RNA level in cells (fold change 155OE to ctrl) and patients (correlation with miR-155) **D.**, NKIRAS1 protein levels were also determined *in vitro* in the control and miR-155 overexpressing cells **E.** cor.: correlation with miR-155 expression.

Combining cell line and patient data sets revealed several genes with coregulation (Figure 6C). The genes present in the table were additionally all validated by qPCR in the cell line. Three genes were consistently strongly deregulated upon stable *in vitro* miR-155 expression and in the miR-155 expressing *IDH* wild type tumors: brain-expressed X-linked protein (BEX)1, atlastin (ATL)1 and tetraspanin (TSPAN)7 (Figure 6C). Yet, these genes were not found to be direct canonical miR-155 targets in different miRNA target predictions (miRTarBase, targetscan, TarBase, miRDB and Pictar). To elucidate the underlying miR-155 molecular pathway effects, transcriptome data was analyzed by Ingenuity Pathway Analysis (IPA) indicating activation of the NFκB pathway upon miR-155 expression (Figure 7A). This was confirmed by immunoblot analysis for the phosphorylation of the NFκB subunit p65, marker for an active canonical NFκB pathway, in the control and miR-155 overexpressing cells (Figure 7B). Moreover, pretreating glioma cells with the NFκB inhibitor JSH-23 specifically reduced therapy resistance in miR-155 overexpressing cells (Figure 7C). Apart from the NFκB inhibitor BEX1 determined above, it was recently published that NFκB inhibitor interacting Ras-like 1 (NKIRAS1) is a direct target of miR-155 [33]. Although the reduction at the RNA level was only marginal in the miR-155 expressing cells, immunoblot analysis showed a strong reduction of NKIRAS1 at the protein level after miR-155 overexpression (Figure 7D, 7E). Moreover, in *IDH* wild type anaplastic glioma patients NKIRAS1 expression was negatively correlated with miR-155 expression at the RNA level (Figure 7D).

## DISCUSSION

In this NOA-04 dataset-based epigenome-wide DNA methylation screen, methylation of the miR-155 promoter was established as strong prognostic marker in patients with anaplastic gliomas in three independent patient cohorts. Mir-55 promoter methylation showed stronger effects than *IDH* mutation status, *MGMT* promoter methylation and histopathology. In functional analyses the onco-miRNA activity of miR-155 was confirmed and the promotion of therapy resistance upon miR-155 expression was associated with increased NFκB activity.

Several recent studies [30–32] have established a classification for anaplastic gliomas consisting of three classes, separable by *IDH* mutation or GCIMP and 1p/19q deletion status as well as presence of absence of copy number variations typical for glioblastoma. Importantly, this molecular classification signifies clinically relevant survival differences and is superior to the current histopathological subtyping [29]. The present study focussed on differentially methylated candidates outside *IDH* and 1p/19q in the non-coding regions of the DNA.

The advantage of methylation biomarkers lays in the easy determination by pyrosequencing / MassARRAY and putative stability over time. In chronic lymphocytic leukemia DNA methylation of a single CpG in the promoter of *ZAP-70* predicts the outcome [34, 35]. Similarly, an HPV-related methylation signature predicts survival in oropharyngeal squamous cell carcinomas [36].

Independent from later miRNA studies already the primary transcript of miR-155, B-cell integration cluster (BIC), was marked as proto-oncogene in chicken B-cell lymphomas induced by the avian leukosis virus [37, 38]. With the upcoming miRNA research, the mature miR-155 was deducted from BIC also in mouse and human [39]. The antisense strand of the miR-155 duplex (miR-155-3p), which is expressed only at minor levels in comparison to miR-155 (miR-155-5p) [40, 41]. miR-155 was found to be overexpressed in leukemia and lymphoma, but also in solid tumors of the lung and breast as well as in glioma [42].

The functional data with transient and stable miR-155 expression modulations are in line with the so far only transient anti-miR-155 siRNA studies. U87MG showed reduced proliferation upon miR-155 inhibition with unmodified siRNAs, as well as a diminished migration and invasion but increased apoptosis [43–45]. Also miR-155 knockdown in U251MG cells resulted in reduced proliferation and enhanced chemo-sensitivity [46]. However, in our hands U251 belonged to the group of cell lines with a high methylation and minimal miR-155 expression levels [43–45].

One initial drawback of the analyses was the existence of the GCIMP in *IDH* mutated gliomas [3, 47], which rendered differential methylation of most attractive candidates in the past just an epiphenomenon of co-methylation despite the potential functional or clinical relevance. Here, we identified a candidate, miR-155, which showed prognostically relevant differential promoter methylation in anaplastic gliomas superior to established marker. This makes miR-155 a particularly attractive candidate since apart from *MGMT* there is no other DNA methylation-regulated candidate marker with relevance outside the *IDH*-effect. The mechanisms, by which MGMT and miR-155 are regulated, remain to be determined [48].

Interestingly, the analysis of TCGA glioblastoma patients, who are mainly *IDH* wild type patients, did not reveal prognostically relevant differential miR-155 promoter methylation (Supplementary Table 3), indicating a marker with some selectivity for anaplastic glioma. The same was seen for malignant gliomas in elderly patients (Supplementary Table 2). This patient group with nearly no *IDH* mutations had been shown to be especially troublesome, since the generally low DNA methylation levels in their tumors prevented proposed gene candidates from being prognostic [49].

One of the earliest onco-miRNA representative is miR-21, whose aberrant expression was first described in a miRNA screen on glioblastoma patient samples yielding miR-21 as the candidate showing strongest upregulation in glioblastoma and demonstrating a functional dependency of glioblastoma cells on its expression [50]. Apart from miR-155 also other miRNAs with promising results in our methylation screen, like miR-210 and miR-335, have been determined as functional onco-miRNA in glioma [51, 52]. Moreover, the TCGA studies on large cohorts of patients with glioblastoma, followed by numerous articles making use of the publically available data, and recently WHO grade II and III glioma highlight the deregulation and involvement of multiple miRNAs species in gliomas [31, 53, 54].

Limitations to our data are the retrospective nature of the analyses as well as the multiple analyses performed on the NOA-04 biomarker data set. To overcome these limitations, data were corrected for multiple testing and stringent criteria were used for the confirmation of the data in independent cohorts. Starting from two unsupervised analyses, candidates were narrowed down by challenging in other datasets and finally cell line experiments. Therefore, it cannot be excluded that though aiming to be comprehensive, we may have omitted candidates that may be of value. Sample size especially with focus on event rates and numbers in the individual subgroups, although considerable in total, may have been too low to decipher predictive effects of miR-155 or any other candidates. As opposed to TCGA and NOA-04, the subpopulation of the GGN cohort investigated in this study did not feature a known distribution of the established biomarker *IDH* mutation and *MGMT* promoter methylation (Supplementary Table 5). The consistent prognostic value of miR-155 promoter methylation in this cohort further underlines its relevance even beyond the clinical study situation (NOA-04) and research oriented patient selection (TCGA).

Besides, the cut-off methylation level of 67% methylation at the miR-155 promoter, applied to split the patients into the “low” and the “high” methylated group, was determined statistically to generate the strongest significance in the NOA-04 cohort (Figure 2). Consequently, although this cut-off value was subsequently confirmed in the GGN cohort (Supplementary Figure 3), further validation and presumably adjustment are necessary, to best serve the actual patient situation. The choice of glioblastoma cell lines instead of cell lines from anaplastic gliomas was made based on the limited availability of manageable WHO grade III cells in culture and the strong effect of miR-155 in *IDH* wild type anaplastic gliomas

Future challenges will be the integration of information of this kind into prospective trials and later into the glioma classification and the present set of clinically applicable biomarkers. Moreover, a proof-of-concept clinical trial is needed to address the value of NFκB or even more distal inhibition in the treatment of patients with miR-155 overexpressing gliomas. Yet, miRNA-based treatments are coming of age and are entering the clinic. Miravirsen, a miR-122 antisense therapy for hepatitis C with hepatitis C virus infection, has recently shown promising results in a phase 2a trial [55]. Additionally, the first miRNA-based cancer therapy MRX34 is in the late stage of a phase I trial on liver cancer and liver metastasis [56]. The blood-brain-barrier constitutes a major hurdle for the treatment of gliomas but already several strategies are pursued with exosome / liposome particles, polymer based vectors and viral systems being at a special focus nowadays [57–59].

For testing of miR-155 promoter methylation levels, the technically easiest and least tissue consuming analyses would be MassARRAY technology or pyrosequencing, both being applicable to formalin-fixed paraffin embedded tumor tissue [19]. Methylation specific PCR is not recommended since it does not generate quantitative results but only produces a binary output: unmethylated or methylated [60].

**Figure 8:**
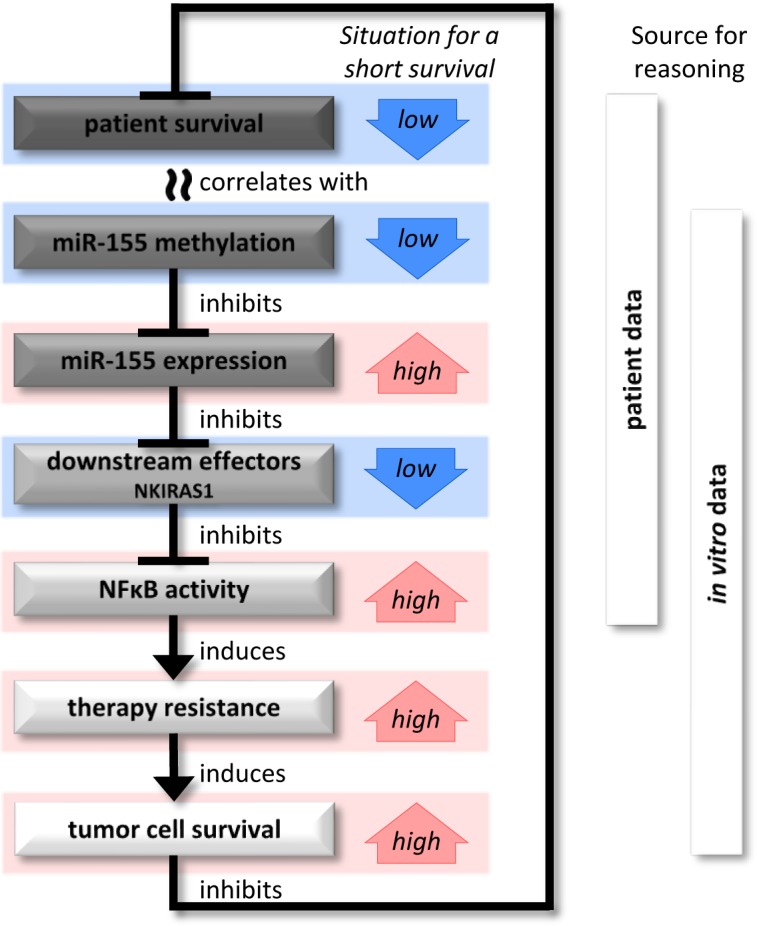
Potential association between patient survival and miR-155 Initially, a short patient survival was correlated with a low DNA methylation at the miR-155 promoter. In the following, patient and *in vitro* data showed that an absence of this inhibitory methylation allowed the expression of miR-155. The expression of miR-155 negatively regulated target mRNAs and led to major expression changes in the cells. In particular, down regulation of NFκB inhibitors enabled the activation of NFκB. This caused an increased resistance against standard clinical therapies and enabled a stronger tumor cell survival, which would explain the short patient survival.

In summary (Figure 8), miR-155 promoter methylation and anaplastic glioma patient survival were correlated and a lower miR-155 promoter methylation was associated with worse prognosis. We further observed that miR-155 promoter methylation and miR-155 expression were negatively correlated, and that high promoter methylation reduced the expression of miR-155. Further, high miR-155 expression was associated with strong transcriptional changes and NFκB pathway activation mediating chemo- and radiotherapy resistance. We hypothesize that these observations explain why patients with anaplastic gliomas featuring high miR-155 expression demonstrate reduced survival.

## MATERIALS AND METHODS

### Patients and tumor samples

Glioma tissue samples were acquired from the two clinical trials NOA-04 and NOA-08 and additionally the prospective cohort study of the German Glioma Network (GGN). The clinically related research in this work is covered by the ethics votes for NOA-04 [6], NOA-08 [15] and the GGN [16] (for molecular characteristics see the supplement).

As opposed to TCGA and NOA-04, the subpopulation of the GGN cohort investigated in this study did not show a known distribution of the established biomarker *IDH* mutation and *MGMT* promoter methylation (Supplementary Table 5).

### Purification of DNA and RNA

DNA from formalin fixed paraffin embedded patient tissue was purified from representative tumor areas with the QIAamp DNA Mini Kit (Qiagen, Hilden, Germany).

DNA together with RNA > 200 bp and RNA < 200 bp was purified from fresh frozen patient tissue and cell lines with the AllPrep DNA/RNA Mini Kit (Qiagen) together with the RNeasy MinElute Cleanup Kit (Qiagen) according protocol to the standard protocol. For samples where DNA was not needed, then miRNAs were purified with the miRNeasy Mini Kit (Qiagen, Hilden, Germany) together with the RNeasy MinElute Cleanup Kit (Qiagen) to obtain an RNA fraction < 200 bp and > 200 bp. For an extraction of only RNA > 200 bp, the RNeasy Kit (Qiagen) was used.

### Methyl-CpG immunoprecipitation (MCIp) and CpG island array hybridization

Methyl-CpG immunoprecipitation (MCIp) with 2.5 μg genomic DNA, array hybridization using CpG island arrays (Agilent, Waldbronn, Germany) and data evaluation were essentially performed as described previously [17]. Enrichment of methylated DNA fragments was controlled by qPCR analyzing the differentially methylated sequence in the gene *PCDHGA11* and on the other hand *GAPDH* as a marker for unmethylated fragments. Differentially methylated regions (DMRs) were defined and identified as described [17]. The array data have been deposited in NCBI's Gene Expression Omnibus under accession number GSE79080.

### MiRNA promoter data and generation of differentially methylated miRNA promoter candidates from data set overlay

For miRNA promoter identification, the H3K4me3 ChIP data set previously published by Baer et al [18] was utilized. These data were generated using an Agilent custom-design 244k array covering genomic loci of 939 annotated miRNAs from miRBase 15 (http://www.mirbase.org/). The sequences represented on the array included 35 kb upstream and 5 kb downstream of the pre-miRNAs and 2 kb up- and downstream of transcriptional start sites of miRNA hosting genes. The array design is available at Agilent's array platform (AMADID029434 for hg19). Analysis of H3K4me3 ChIP data was performed with the CoCAS ChIP-on-chip analysis suite as described previously [18]. In total, 781 putative miRNA promoter regions were identified. For detection of overlaps of genomic features (differentially methylated regions and putative miRNA promoter regions) a custom PERL script based on pair wise comparison of the genomic coordinates was used (script is available on demand).

### MassARRAY DNA methylation analysis

DNA of formalin fixed paraffin embedded patient tissue was bisulfite converted using the Epitect Bisulfite Kit (Qiagen). DNA methylation was quantitatively measured with the MassARRAY system (Sequenom) as previously described [19]. Primer sequences are listed in the supplementary material.

### *In vitro* work

LN-308, LN-319, LN-428, D247 and U373 glioma cells were kindly provided by Prof. Nicolas de Tribolet (Lausanne, Switzerland). LN-229, LN-18, U138 MG, T98G, A172 and U87MG cells were purchased from the American Type Culture Collection (ATCC; Manassas, VA, USA). Regular checks for cell line authenticity and freedom from infection, e.g. mycoplasms, were done according to the institutional guidelines at the German Cancer Research Center.

For inhibition of DNA methylation by 5-aza-2′-deoxycytidine (decitabine) cells were seeded in 6-well plates with two wells for each condition. The cells were incubated with 0,5μM decitabine or DMSO as control treatment and the medium was exchanged every day. T98G, U251 and U138 were treated three days and A172 and U87MG for five days.

MiR-155 (miR-155-5p) specific siRNA and control siRNA were purchased from Exiqon (Vedbaek, Denmark; miRCURY LNA Power inhibitors). These siRNAs are based on the Locked Nucleic Acid (LNA) technology from Exiqon and feature a fully phosphorothioate (PS) modified backbone. For stable miR-155 overexpression the glioma cell line T98G was transfected with the pre-miR-155 containing episomal vector pREP4 (Invitrogen, Carlsbad, CA, USA) for miR-155 overexpression and the prep4 backbone as control. The ready-to-use vector was kindly provided by Jan Meier [20]. Further details on transfection procedures and assay procedures are found in the supplement.

### Quantitative reverse transcription polymerase chain reaction

The miRNA fraction was reversely transcribed with the Universal cDNA Synthesis Kit II (Exiqon A/S, Vedbaek, Denmark) and the expression then analyzed with the miRCURY LNA™ Universal RT microRNA PCR Kit and respective primer (Exiqon) on a StepOnePlus Real-Time PCR System (Applied Biosystems, Carlsbad, CA, USA).

miR-16 was established as reference with BestKeeper [21] analysis due to is high reproducibility and low variance after the evaluation of miR-16, U6 snRNA, SNORD48, 5S RNA and let7a. Moreover, using RNA species longer than miRNAs, e.g. U6 snRNA, was not recommended due to potential differences in extraction and reverse transcription (e.g. see Exiqon miRNA qPCR guidelines). Besides, retesting the effect of decitabine demethylation using U6 as reference gene resulted in comparable effects for miR-155 expression.

RNA was transcribed with the High-Capacity cDNA Reverse Transcription Kit (Applied Biosystems, Warrington; UK) and the ABsolute qPCR SYBR Green Mix (Thermo Scientific, Waltham, USA) used for expression analysis relative to GAPDH. Primer sequences are listed in the supplement.

The miRNA name refers to this form throughout the paper (miR-155 for miR-155-5p, miR-210 for miR-210-3p and miR-335 for miR-335-5p).

### Immunoblot

Cell lysates and immunoblots were performed as described previously [22]. Antibodies: α-Tubulin 1:1000 (#3873; Cell Signaling, Cambridge, UK), β-actin 1:1000 (sc-1616; Santa Cruz, Dallas, Texas, USA), p-NF-κB p65 (L8F6) 1:1000 (#3033; Cell Signaling, Cambridge, UK); NKIRAS1 1:1000 (GTX113962; Genetex, Irvine, CA, USA).

### TCGA collective

Methylation (Illumina HumanMethylation450 BeadChip), miRNA expression (miRNASeq), mutational (*IDH*) and survival data have been obtained from The Cancer Genome Atlas dataset at April 15^th^, 2013 (http://cancergenome.nih.gov).

### Microarray analysis

Cells were treated as described for the proliferation assay and prepared as described before [22]. The microarray data have been deposited in NCBI's Gene Expression Omnibus under accession number GSE79081. A detailed description is found in the supplement.

### Statistical analysis

MassARRAY CpG units were evaluated separately as well as averaged per amplicon. Association of quantitative DNA methylation measurements with overall survival (OS) and progression-free survival (PFS) was assessed with univariable Cox PH regression models. Proportional hazards assumption was tested for violation according to Grambsch and Therneau [23]. Risk groups were determined based on optimal cut-point analysis using the maximally selected log-rank statistic approach, which corrects for type I error inflation due to multiple testing [24]. Survival of risk groups was estimated with the method of Kaplan and Meier. Univariable p-values were adjusted for multiple testing across all amplicons using Benjamini-Hochberg correction in order to control the false discovery rate [25]. Multivariable Cox regression was used to adjust for clinico-pathological factors. All tests were two-sided. P-values below 0.05 were considered statistically significant. Analyses were carried out using software R Version 2.14 using add-on package rms [26].

For the TCGA data, correlation of methylation and expression was analyzed per cg probe with Pearson's correlation coefficient. Illumina cg probes were selected to cover the MassARRAY amplicon. For methylation data, the M value = log2 (Methylated Intensity+1) / (Unmethylated Intensity+1) was used, for miRNA expression normalized counts (reads per million miRNA mapped). Survival analysis employed Cox proportional hazards regression and the log-rank test with maximally selected rank statistics (R package “maxstat”), which corrects for Type-I-error inflation [24]. Violation of the proportional hazards assumption was tested according to Grambsch and Therneau [23]. To adjust for type-I-error inflation, the Bonferroni procedure was used. All tests were two-sided, and a *p* value < 0.05 was considered significant. Analyses were carried out using R version 3.0.2 [27].

The *in vitro* experiments were conducted in at least three independent experiments if not otherwise indicated. Two-sided Student's t test was applied for statistical evaluation. Values *p* < 0.05 were considered significant and marked with asterisks. All error bars shown in figures represent standard deviation.

## SUPPLEMENTARY MATERIALS AND METHODS


