# Supplementary material for: Prognostic relevance of miRNA-155 methylation in anaplastic glioma
Source: Oncotarget. 2016 Nov 18;7(50):82028–45. doi: 10.18632/oncotarget.13452 (PMC5347671; doi:10.18632/oncotarget.13452)
Supplement: Supplementary file 1 [file oncotarget-07-82028-s001.pdf]

Prognostic relevance of miRNA-155 methylation in anaplastic glioma

Supplementary Materials and Methods

Patient characteristics of the anaplastic glioma NOA-04 trial patients (n=NN) analyzed in this paper.

		number of patients	ratio
***MGMT* promoter**	methylated	63	60%
	unmethylated	42	40%
IDH	wild type	27	26%
	mutant	76	74%
1p/19q	codeletion	39	42%
	non-codeletion	53	58%
**Histology**	astrocytic	50	48%
	oligoastrocytic	37	35%
	oligodendroglial	18	17%
**1st line therapy**	chemotherapy	53	50%
	radiotherapy	52	50%

Patient age (mean years at operation) 44.8

Patient characteristics of the anaplastic glioma GGN cohort patients (n=NN) analyzed in this paper. Patients with oligodendroglioma and oligoastrocytoma were combined in the oligodendroglial category.

		number of patients	ratio
***MGMT* promoter**	unmethylated	12	17%
	methylated	60	83%
IDH	wild type	23	31%
	mutant	52	69%
**Histology**	astrocytic	37	45%
	oligodendroglial	45	55%
**Therapy**	none	6	7 %
	chemotherapy	10	12%
	radiotherapy	22	27%
	chemo- and radiotherapy	44	54%

Characteristics of the astrocytoma NOA-08 trial patients (n=NN) analyzed in this paper.

		number of patients	ratio
***MGMT* promoter**	unmethylated	66	64%
	methylated	37	36%
IDH	wild type	90	99%
	mutant	1	1%
**Histology**	anaplastic astrocytoma	6	6%
	glioblastoma	100	94%
**Therapy**	radiotherapy	55	52%
	chemotherapy	56	48%

In the different assays, patient numbers may vary for technical reasons. There was no other bias preventing the same number of tissues from being analyzed and the maximal attempt made to ensure the highest possible completeness.

MassArray Primer

primer	forward primer	reverse primer	annealing temp.
miR-10b	GATTTTGGTAGAAGAATGAGGGAAT	ATCCCACCCAAAATAAAATACCTAA	56
miR-10b (b)	TTTTTTTTAGATTGGTAGTGGAAGT	CCCCACACAAAATTCAAATTACTAC	56
miR-22	GGTGGTTGGGTAGTTTTTTTAGG	CCCCCTCCCTAAACCATACTAC	56
miR-22 (b)	TATATTTATTTGGTGTTGGGTGTGG	AAAAATAACCTCCTTACCAATCAAAA	56
miR-34bc	GAGAGAGTTAGTTTTAGGGTTTGGG	AAAAATACCAAACCTCCCCTTC	56
miR-96, -182, -183	GAATGGGGATAAGGGGTTTTTA	CTCCCTCTCTTAAAACAAACAACCT	56
miR-96, -182, -183 (b)	GTTGAGAGTTGGGTTTGGGGTA	ACCCTCTACCTATCACCCAATAAAC	56
miR-129-1	TTTAAGTTGTAGTGTAGTGGTGTGAT	AAAACCAACCTAAACAACATAACAA	56
miR-129-1 (b)	ATTTGGTTAGGTTGTAGGGATAGTG	ACAATAAAAAACAAACAAAAAAACC	52
miR-132, -212	GGGTAGTAAGTAGTTTAGAGTTAAGGTG	AAAATACCCCCTCTAAAACATCT	56
miR-155	GAATAAAGGTTGGAGTTTAAGTTTTG	CCTACCTATTCTTAAAACCTACAAC	56
miR-155 (b)	GTTTTTTGGGGATTTAGTGGTGT	CACTCCAACAAAAAAACTAATCCAA	56
miR-195, -497	GGGAGTTTATTTTTTTGGATTTTTA	TTTCTAATTAATTTCCTTCCATTATCC	52
miR-200a+b, -429	TTTTATTTTAGTTTGTGTAGGTGGGA	AATCCAAAAATAAATCAAAAAACCC	52
miR-210	GAAGTTTTTGGAGTTTGGAGGG	CCTTTAACTACCACTTTAAAACCCC	56
miR-335	TATGGAGTTTTGTAGGTAAGGTTTT	CCTACCCCTAAAACTAATATAACCCA	52
miR-335 (b)	GTTTATTTGAGGAGGGGGTGTTAT	AATACCAAAATCTAAAAATCCCAATTC	56
miR-1305	TGTTGTAGTGTTTATTAGGGGTTGG	ACATTTCAAATCAACTAAATCACCC	56

qPCR Primer

gene	forward primer	reverse primer
GAPDH	CTCTCTGCTCCTCCTGTTCGAC	TGAGCGATGTGGCTCGGCT
ABCC2	TCCTACAGTGCTCTCCTGGC	CAGCCCATAGTCATCGTCTTC
ASB5	AACCCAAGGACAAGGTTCC	ACATGGTCTAAGGTTACTGCA

ATL1	TGTAACATGGATTTGGGATGTGG	TTGGCTACTTAGTCCCGAGAG
BEX1	TCATAGTCTGCGGGCAGTCA	AACCATCAGGATTCAGG
BIRC3	GGGAAGAGGAGAGAGAAAGAGC	TCCGGCAGTTAGTAGACTATCCA
CAV2	AGTTCCTGACGGTGTTCCTG	GCACTGAAGGCAGAACCATT
CCDC102A	TCCAGCATGGACCGGAAAATG	CCGCAGCTTCTTGTTCTCC
CDH11	CTGACTTGTGAATGGGACCG	CGCTGAGCTGAAAACACAGT
COL5A2	CCAGGAAGAAGACGAGGATG	AGAATGGCTCCATTGTCACAG
CPA4	TCTGTGTCGGGCACTGAGTA	CAGGAGGAAGCCATAGGTCC
CREG1	GGCGTGCCCTATTTCTACCT	AAAGGGGACTTTGTGGATCA
EFEMP1	CAGCAGGCTACGAGCAAAGT	GATATCCAGGAGGGCACTGA
FAM65C	ATGCCTCCTGGAAGAAGTG	GTGGGATGATCTCTTCAATGG
GPNMB	CGTGAGAATTCAGCATGGAA	TGCTCCCTCATGTAAGCAGA
GPX3	GCCGGGGACAAGAGAAGT	GAGGACGTATTTGCCAGCAT
LAYN	GAGGCGTGAGGAGAAACAAA	GATGGTACATGACCACGCAG
LOC645381	AACATCAACAACAGGTGGCC	GGTCCGTGGCCATAAGAAAG
MAF	ACGAGAAGTTGGTGAGCAGC	TTCCAAAATGTGGCGTATCC
MMP3	GGCCAGGGATTAATGGAGAT	CAAAGCTTCAGTGTTGGCTG
NHS	ACATCCAGCTCACCCACC	GCGCGGTAGTACACACTCAG
STOM	ACGGTGAATAAGAATGAGAACG	CTGAGGAATGTTCTGGGCA
TMEM156	CACCGAAAGAAAGAACATTGG	TTCACCTGTGATGTCTTGG
TNFRSF19	CTCCTCCTCCTTACGAACCG	TGACACAGAGGATGAGCAGG
TSPAN7	GCATCGAGGAGAATGGAGAC	GTGCCCAGAGTAAGTTTGCC
TXNIP	CTTCGGAGTACCTGCGCTAT	TTGAAGGATGTTCCCAGAGG
VGLL4	ATCTCTGGTGCATGCTGATG	CTGAACTTCCTCTTGCTGGG

siRNA Transfection and Functional Experiments

The siRNAs (miRCURY LNA Power inhibitors) from Exiqon (Vedbaek, Denmark) were transfected using Lipofectamine RNAiMAX system (Invitrogen, Carlsbad, CA, USA).

For the viability assay cells were seeded in black Nunc 96-well plates (Thermo Fisher Scientific Inc., Waltham, MA, USA) with 3 wells per condition (U87 1,000 cells/well; LN-428 700 cells/well and A172 500 cells/ well) and 24 h later transfected with the siRNA at 20 nM and 40 nM. 6 days after siRNA transfection medium was exchanged with medium containing 10% (v/v) AlamarBlue and the fluorescence measured 3.5 h later. miR-155 activity was measured with the psiCheck2 system reporter system (Promega, Mannheim, Germany) containing a renilla luciferase with perfect miR-155 binding site in the 3’ UTR and an independent firefly luciferase. Additionally, a psiCheck2 system was transfected as control that contained a renilla and firefly luciferase without miR-155 binding site. 1 day after seeding in 24-well plates (U87MG with 25,000 cells/well; LN-428 and A172 with 15,000 cells/well) were transfected at 10 ng/µl using the TransIT transfection system (Mirus Bio LLC, Madison, WI, USA).

Stable miR-155 Expression and Functional Experiments

The transfection of the prep4 plasmids was conducted with TransIT according to the manufacturer’s recommendations and then the cells selected and cultured in 200 µg/ml

Hygromycin (Invitrogen) containing DMEM (Sigma-Aldrich, St. Louis, MO, USA) with 10% FBS (Sigma-Aldrich) and 1% PenStrep (Sigma-Aldrich).

For the proliferation analysis cells were seeded at 40.000 cells/well in 6-well plates. 24 h later they were treated daily for 72 h with 300 µM TMZ (Sigma-Aldrich). At the fourth day the plates were irradiated with 4 Gy (Gammacell 40 Exactor, Best Theratronics, Ottawa, Canada). The next day all cells were harvested and seeded at 3000 cells/well in duplicates and the proliferation measured with the xCelligence Real-Time Cell Analyzer System (RTCA) (Roche Diagnostics, Mannheim, Germany).

For the colony formation assay cells were seeded at 500 cells/well in triplicates in 6-well plates and after 24 h treated. For chemotherapy, the cells were treated daily for 48 h with 300 µM TMZ containing medium. For radiotherapy the plates were irradiated with 4 Gy. 9 days after seeding the cells were fixed and stained (0,5 % crystal violet, 20 % methanol) and colonies counted. The NFκB inhibitor JSH-23 (Merck Millipore, Billerica, USA) was added at a concentration of 5 µg/ml 4 h after seeding and during the TMZ treatment or till 24h after radiation and then replaced with fresh medium.

Microarray Analysis

RNA quality check

The quality of total RNA was checked by gel analysis using the total RNA Nano chip assay on an Agilent 2100 Bioanalyzer (Agilent Technologies GmbH, Berlin, Germany). Only samples with RNA index values greater than 8.5 were selected for expression profiling. RNA concentrations were determined using the NanoDrop spectrophotometer (NanoDrop Technologies, Wilmington, DE).

Probe Labeling and Illumina Sentrix BeadChip array Hybridization

The laboratory work was done in the Genomics and Proteomics Core Facility at the German Cancer Research Center, Heidelberg, Germany (DKFZ). Biotin-labeled cRNA samples for hybridization on Illumina Human Sentrix-8 BeadChip arrays (Illumina, Inc.) were prepared according to Illumina's recommended sample labeling procedure based on the modified Eberwine protocol [1]. In brief, 250 – 500 ng total RNA was used for complementary DNA (cDNA) synthesis, followed by an amplification/labeling step (in vitro transcription) to synthesize biotin-labeled cRNA according to the Illumina® Total Prep™ RNA Amplification Kit (Life Technologies). Biotin-16-UTP was purchased from Roche Applied Science, Penzberg, Germany. The cRNA was column purified according to TotalPrep RNA Amplification Kit, and eluted in 60-80 µl of water. Quality of cRNA was controlled using the RNA Nano Chip Assay on an Agilent 2100 Bioanalyzer and spectrophotometrically quantified (NanoDrop).

Hybridization was performed at 58°C, in GEX-HCB buffer (Illumina Inc.) at a concentration of 100 ng cRNA/µl, unsealed in a wet chamber for 20 h. Spike-in controls for low, medium and highly abundant RNAs were added, as well as mismatch control and biotinylation control oligonucleotides. Microarrays were washed once in High Temp Wash buffer (Illumina Inc.) at 55°C and then twice in E1BC buffer (Illumina Inc.) at room temperature for 5 minutes (in between washed with ethanol at room temperature). After blocking for 5 min in 4 ml of 1% (wt/vol) Blocker Casein in phosphate buffered saline Hammarsten grade (Pierce

Biotechnology, Inc., Rockford, IL), array signals are developed by a 10-min incubation in 2 ml of 1 µg/ml Cy3-streptavidin (Amersham Biosciences, Buckinghamshire, UK) solution and 1% blocking solution. After a final wash in E1BC, the arrays are dried and scanned.

Scanning and data analysis

Microarray scanning was done using an iScan array scanner. Data extraction was done for all beads individually, and outliers were removed when the absolute difference to the median was greater than 2.5 times MAD(2.5 Hampel's method). All remaining bead level data points were than quantile normalized [2]. As test for significance the student's t-test was used on the bead expression values of the groups of interest. In the case of significance of expression against background we tested for greater than all negative beads for this sample and in the case of comparing separate groups we tested for inequality of the means of the groups. Benjamini-Hochberg correction [3] was applied to the set of preprocessed ProbeIDs. The average expression value was calculated as mean of the measured expressions of beads together with the standard deviation of the beads.

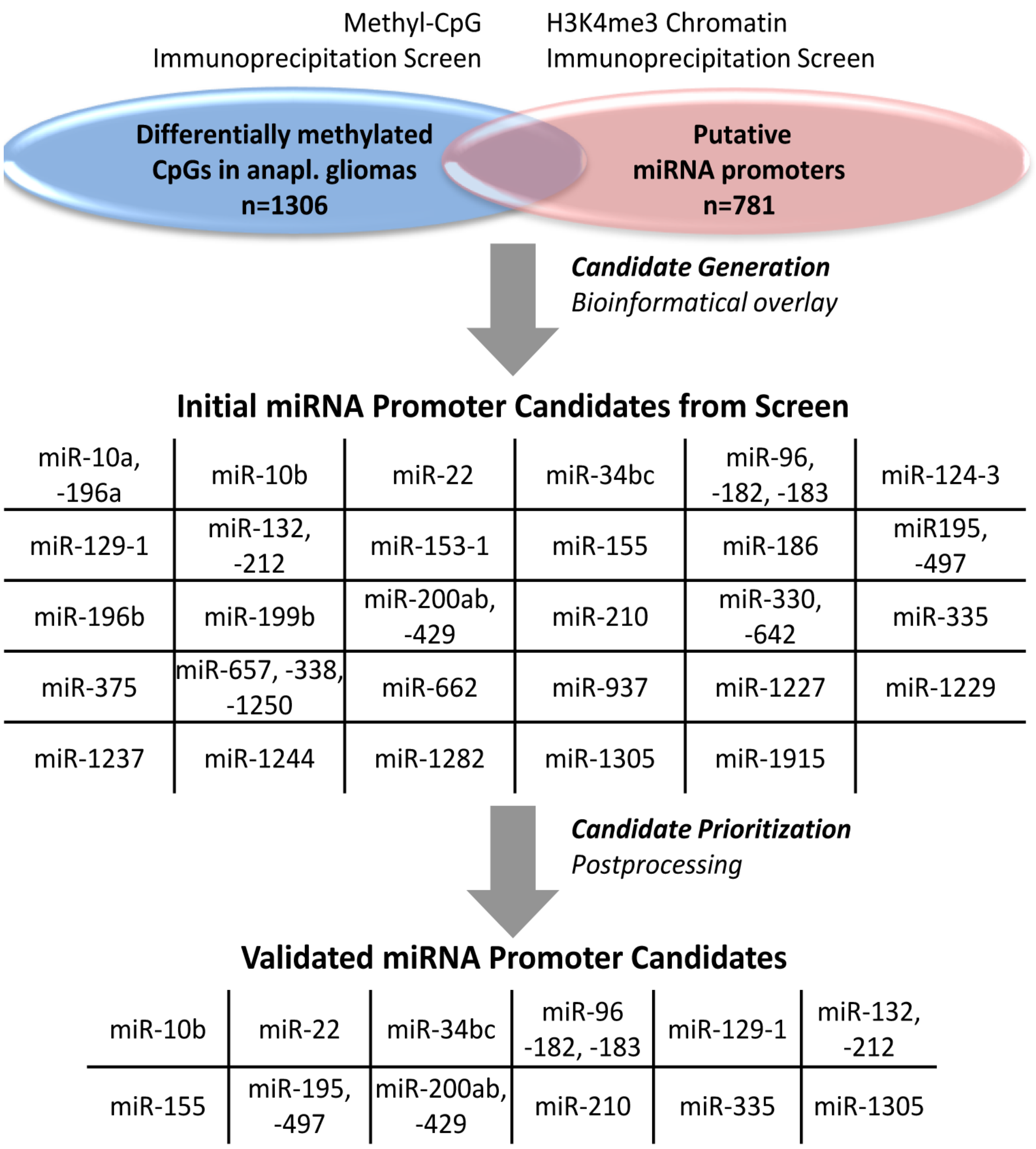

Candidate Generation
Bioinformatical overlay

Initial miRNA Promoter Candidates from Screen

miR-10a, -196a	miR-10b	miR-22	miR-34bc	miR-96, -182, -183	miR-124-3
miR-129-1	miR-132, -212	miR-153-1	miR-155	miR-186	miR195, -497
miR-196b	miR-199b	miR-200ab, -429	miR-210	miR-330, -642	miR-335
miR-375	miR-657, -338, -1250	miR-662	miR-937	miR-1227	miR-1229
miR-1237	miR-1244	miR-1282	miR-1305	miR-1915	

Candidate Prioritization
Postprocessing

Validated miRNA Promoter Candidates

miR-10b	miR-22	miR-34bc	miR-96 -182, -183	miR-129-1	miR-132, -212
miR-155	miR-195, -497	miR-200ab, -429	miR-210	miR-335	miR-1305

Supplementary Fig. 1 miRNA candidates were generated by the overlay of two data sets and prioritization. The initial list of differentially methylated miRNA promoter candidates in anaplastic (anapl.) glioma originated from an overlay of two distinct screens: a methyl-CpG immunoprecipitation screen for differentially methylated regions in anaplastic glioma from the NOA-04 trial (n = 4; healthy n = 1) *vs.* an H3K4me3 chromatin immunoprecipitation data set from cell lines of different origin (n = 6) and chronic lymphocytic leukemia patients (n = 24; healthy n = 10) for putative miRNA promoters. From the 29 candidates produced by this overlay the 12 most favorable candidate regions were validated by MassArray

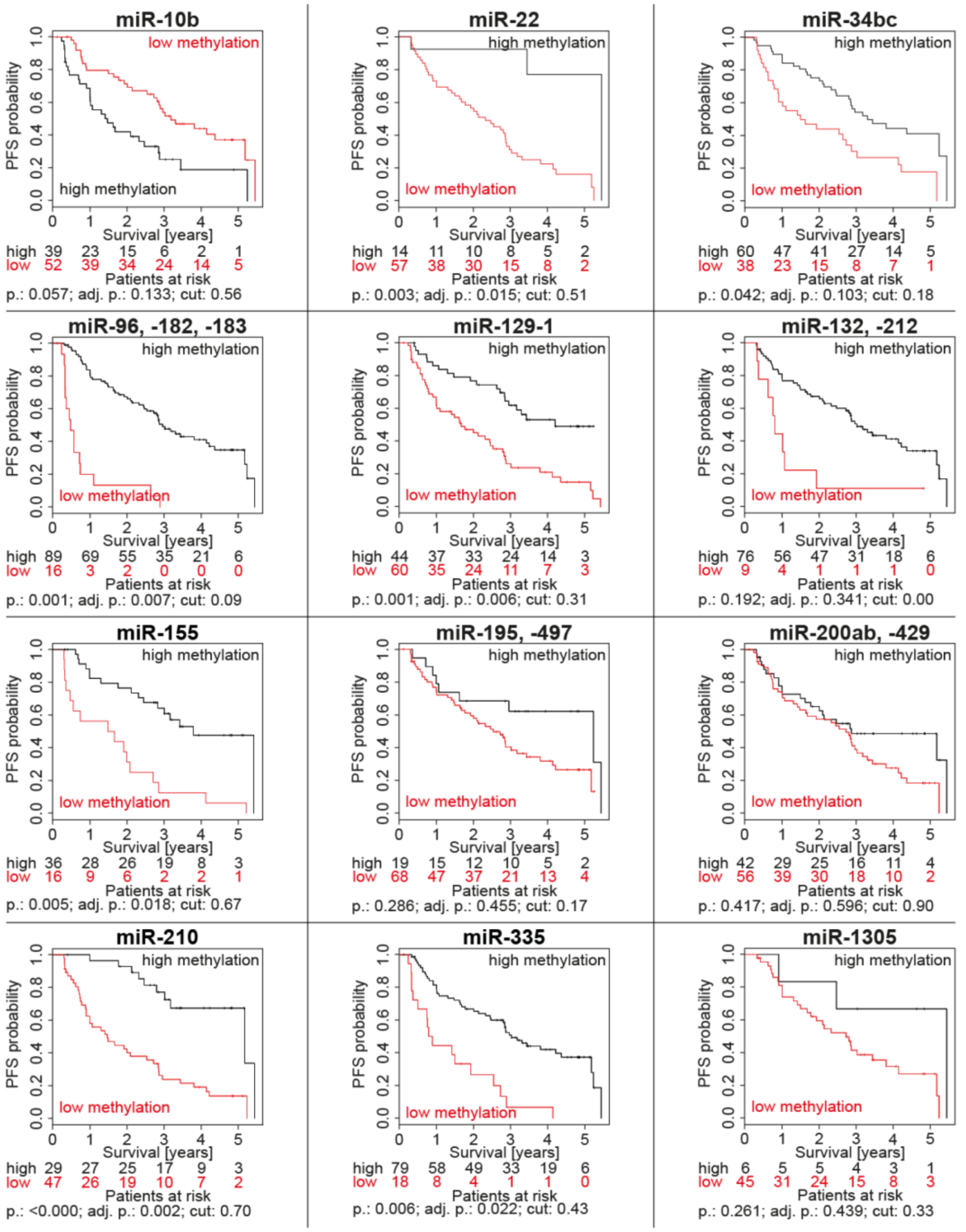

Supplementary Fig. 2 Prognostic miRNA methylation was determined for patients with anaplastic gliomas of the NOA-04 trial - by Kaplan-Meier estimate. Kaplan-Meier curves calculated from the mean methylation levels of the miRNA candidates are shown. The statistically determined cutpoint depicts the threshold separating the patients in low and high methylation. The number of patients in the each group is listed below each graph. PFS: progression free survival; p.: individual p-value; adj. p.: p-value adjusted for testing of multiple amplicons, CpG fragments and cutpoints

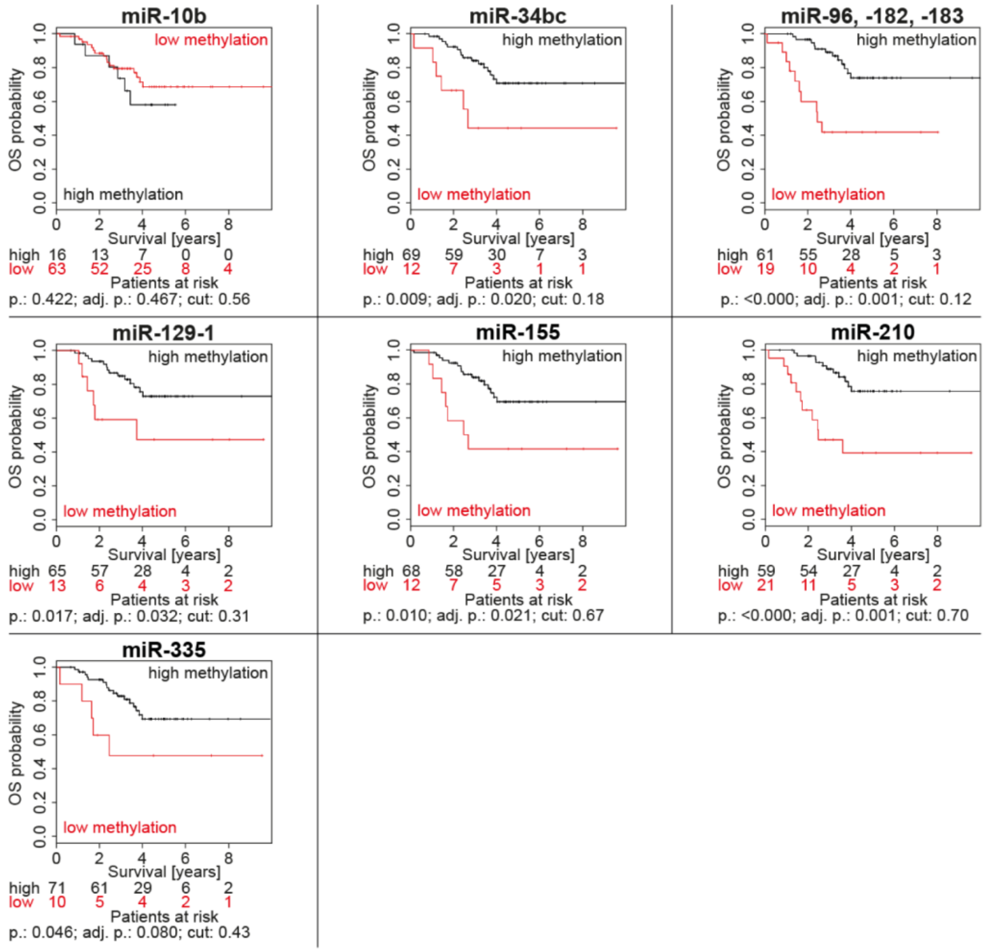

Supplementary Fig. 3 Prognostic miRNA methylation was validated in patients with anaplastic gliomas of the German Glioma Network (GGN) - by Kaplan-Meier estimate. Mean methylation levels of the miRNA candidates were used. Cut points determined from the NOA-04 patients were applied. The number of patients in the each group is listed below each graph. OS: overall survival; p.: individual p-value; adj. p.: p-value adjusted for testing of multiple amplicons, CpG fragments and cutpoints

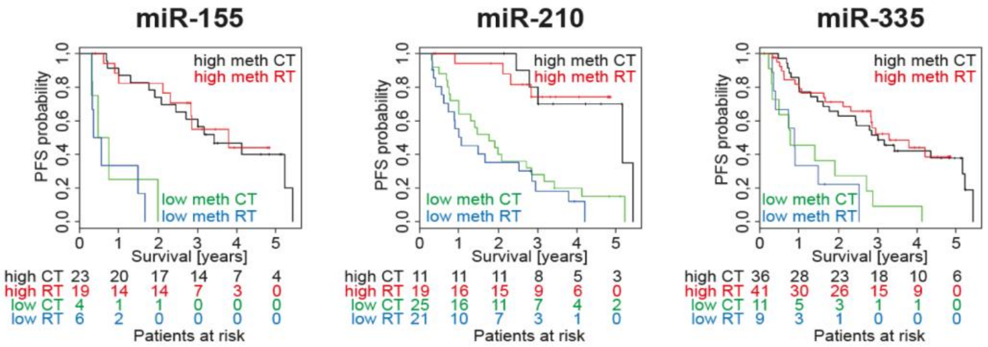

Supplementary Fig. 4 Survival advantages of miRNA methylation levels were independent of initial therapy. The patients from the NOA-04 trial were further separated according to the initial therapy (chemo- or radiotherapy). The methylation of the candidate regions was then correlated for these treatment groups with the progression free survival by Kaplan-Meier regression model. Shown are the results for the three main candidates. PFS: progression free survival; CT: chemo therapy; RT: radio therapy

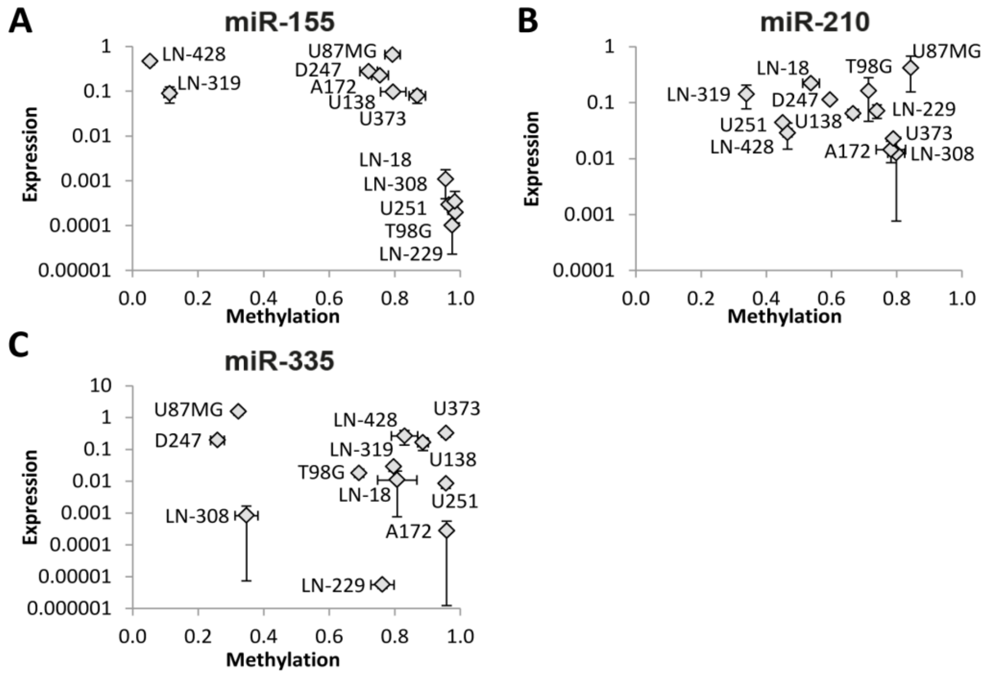

Supplementary Fig. 5 miR-155 methylation and expression were negatively correlated in glioma cell lines. 12 established glioma cell lines were tested for their miRNA methylation by MassArray and expression by qPCR. Results for the three main candidates from two independent replicates are shown: miR-155 (a; p=0.034), miR-210 (b; p=0.590) and miR-335 (c; p=0.555)

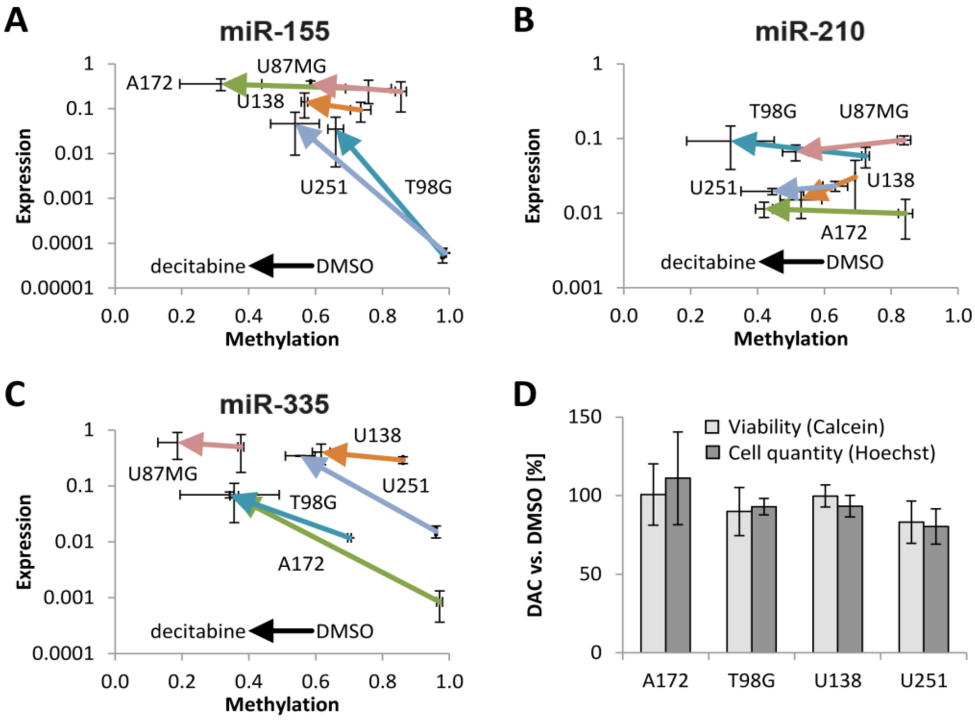

Supplementary Fig. 6 Demethylation by decitabine led to upregulation of miR-155 and miR-335. Cells were treated with 5-aza-2'-deoxycytidine (decitabine) to demethylate the miRNA promoter region of miR-155 (A), miR-210 (B) and miR-335 (C). Methylation and expression was measured of mock (DMSO) treated and decitabine treated cells in two independent experiments. In parallel also cell viability and cell count was monitored once (D)

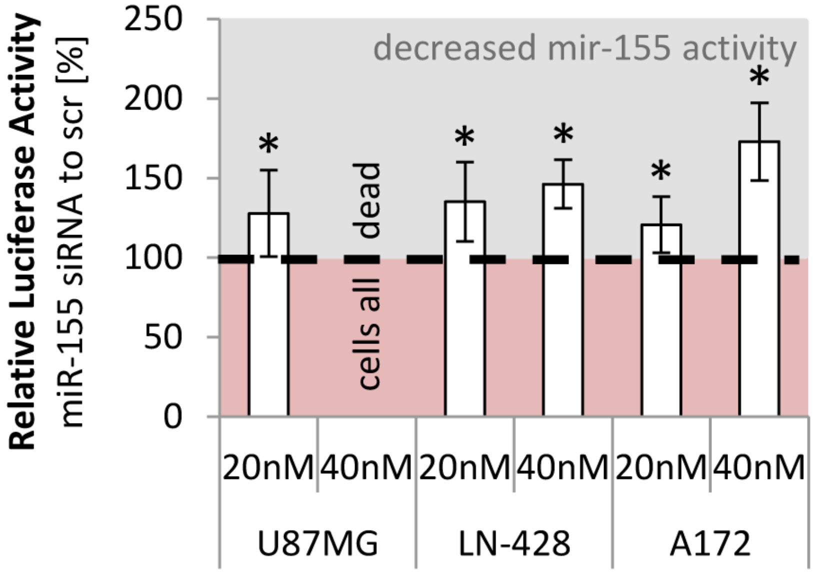

Supplementary Fig. 7 Transient siRNA miR-155 knockdown. miR-155 high expressing glioma cell lines (U87MG, LN-428 and A172) were transfected with scrambled (scr) and anti-miR-155 siRNA (si155) in two concentrations. To confirm a functional miR-155 knock down cells were additionally transfected with a luciferase reporter system to measure miR-155 activity. p-values < 0.05 are marked by an asterisk

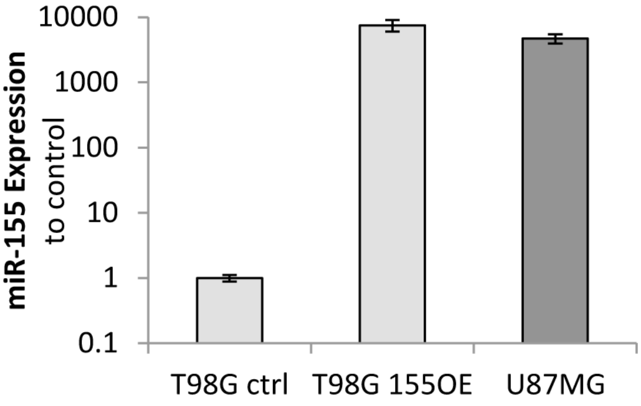

Supplementary Fig. 8 miR-155 was stably overexpressed in T98G. miR-155 was overexpressed in the glioma cell line T98G by transfecting the pREP4 plasmid encoding the pri-miRNA of miR-155. miR-155 expression levels were measured by qPCR. Due to the nominal expression in wild type T98G a three log level overexpression was achieved, which was close to wild type miR-155 expression levels in U87MG. ctrl: control transfection; 155OE: miR-155 overexpression

Supplementary Table 1a-c Results from all analyzed amplicons and CpG fragments by Cox regression and Kaplan-Meier estimate from the methylation analysis on the NOA-04 and GGN patients. For several miRNA candidates the methylation in a second region was measured, which were marked by (b). Significant correlations were denoted in grey (light gray p-value < 0.05; dark gray adj. p-value < 0.05). Obs: observation, number of patients with full data; HazR: Hazard ratio; CI: confidence interval; p-value: individual p-value; p-value adj: p-value adjusted for testing of multiple amplicons, CpG fragments and cutpoints; cut: statistically determined optimal cutpoint to separate the patients into the low and high methylation groups

Supplementary Table 1a

NOA-04		Progression Free Survival								Overall Survival							
		Cox regression					Kaplan-Meier estimate			Cox regression					Kaplan-Meier estimate		
		Obs	HazR	95% CI	p-value	**p-value adj**	cut	cut p-value	**cut p-value adj**	Obs	HazR	95% CI	p-value	**p-value adj**	cut	cut p-value	**cut p-value adj**
miR-10b	CpG_1	91	1.35	[0.82, 2.24]	0.237	**0.393**	0.81	0.118	**0.243**	91	1.13	[0.61, 2.07]	0.705	**0.805**	0.92	0.848	**0.936**
	CpG_2.3.4	91	1.27	[0.82, 1.96]	0.287	**0.440**	0.82	0.350	**0.526**	91	1.09	[0.64, 1.85]	0.758	**0.842**	0.60	0.839	**0.936**
	CpG_5	75	1.36	[0.78, 2.38]	0.283	**0.440**	0.98	0.370	**0.546**	75	1.62	[0.79, 3.33]	0.186	**0.386**	0.65	0.261	**0.454**
	CpG_6	91	1.86	[1.23, 2.79]	0.003	**0.011**	0.15	0.023	**0.063**	91	1.67	[1.01, 2.77]	0.045	**0.125**	0.44	0.086	**0.209**
	mean	91	1.60	[1.05, 2.45]	0.029	**0.080**	0.56	0.057	**0.133**	91	1.42	[0.84, 2.39]	0.191	**0.386**	0.66	0.239	**0.447**
miR-10b (b)	CpG_1	99	1.02	[0.76, 1.36]	0.920	**0.947**	0.04	0.645	**0.829**	99	0.99	[0.68, 1.44]	0.959	**0.959**	0.07	0.925	**0.949**
	CpG_9.10	99	0.78	[0.50, 1.20]	0.258	**0.421**	0.01	0.117	**0.243**	99	0.64	[0.36, 1.13]	0.126	**0.283**	0.04	0.173	**0.362**
	CpG_11.12	96	0.58	[0.37, 0.91]	0.018	**0.053**	0.18	0.019	**0.053**	96	0.52	[0.30, 0.92]	0.025	**0.074**	0.18	0.023	**0.070**
	mean	99	0.70	[0.44, 1.10]	0.122	**0.238**	0.14	0.020	**0.056**	99	0.61	[0.34, 1.09]	0.094	**0.219**	0.14	0.039	**0.104**
miR-22	CpG_1.2	71	0.34	[0.19, 0.59]	0.000	**0.001**	0.61	0.000	**0.004**	71	0.28	[0.13, 0.59]	0.001	**0.004**	0.61	0.003	**0.015**
	CpG_3	71	0.45	[0.26, 0.79]	0.005	**0.019**	0.04	0.004	**0.018**	71	0.26	[0.11, 0.61]	0.002	**0.009**	0.04	0.001	**0.009**
	CpG_4	71	0.88	[0.61, 1.27]	0.499	**0.649**	0.21	0.525	**0.698**	71	0.76	[0.46, 1.27]	0.293	**0.561**	0.04	0.301	**0.506**
	CpG_5	69	0.56	[0.33, 0.94]	0.027	**0.077**	0.17	0.041	**0.101**	69	0.54	[0.27, 1.08]	0.079	**0.192**	0.02	0.212	**0.407**
	CpG_7	70	0.85	[0.64, 1.13]	0.268	**0.421**	0.11	0.197	**0.345**	70	0.75	[0.49, 1.15]	0.191	**0.386**	0.25	0.435	**0.649**
	mean	71	0.45	[0.28, 0.73]	0.001	**0.005**	0.51	0.003	**0.015**	71	0.32	[0.16, 0.64]	0.001	**0.005**	0.30	0.004	**0.021**
miR-22 (b)	CpG_2.3	33	0.83	[0.57, 1.20]	0.322	**0.463**	0.06	0.419	**0.596**	33	0.98	[0.66, 1.45]	0.914	**0.929**	0.05	0.736	**0.895**
	CpG_9	33	1.00	[0.83, 1.20]	0.971	**0.987**	0.07	0.735	**0.885**	33	1.02	[0.79, 1.32]	0.856	**0.898**	0.07	0.866	**0.936**
	CpG_10	33	1.01	[0.90, 1.13]	0.912	**0.947**	0.04	0.749	**0.885**	33	0.90	[0.71, 1.14]	0.397	**0.632**	0.04	0.180	**0.369**
	CpG_11.12.13	32	0.98	[0.87, 1.11]	0.783	**0.861**	0.02	0.768	**0.885**	32	0.90	[0.72, 1.13]	0.377	**0.616**	0.01	0.096	**0.223**
	CpG_16	33	1.01	[0.94, 1.08]	0.781	**0.861**	0.00	0.484	**0.658**	33	0.95	[0.78, 1.14]	0.563	**0.702**	0.08	0.647	**0.842**
	CpG_17.18	33	1.05	[0.88, 1.25]	0.595	**0.712**	0.06	0.870	**0.916**	33	0.96	[0.73, 1.27]	0.785	**0.854**	0.01	0.149	**0.322**
	CpG_19	33	0.99	[0.89, 1.10]	0.862	**0.919**	0.07	0.944	**0.975**	33	0.88	[0.60, 1.29]	0.512	**0.702**	0.01	0.797	**0.919**
	CpG_20.21	33	1.07	[0.89, 1.29]	0.455	**0.598**	0.05	0.507	**0.681**	33	1.08	[0.91, 1.29]	0.374	**0.616**	0.05	0.410	**0.628**
	CpG_22.23	33	0.97	[0.85, 1.11]	0.651	**0.765**	0.05	0.799	**0.903**	33	1.02	[0.90, 1.16]	0.723	**0.817**	0.00	0.572	**0.769**
	mean	33	0.97	[0.57, 1.66]	0.923	**0.947**	0.04	0.834	**0.916**	33	0.69	[0.32, 1.48]	0.337	**0.599**	0.07	0.665	**0.857**
miR-34bc	CpG_1	96	0.74	[0.53, 1.03]	0.075	**0.173**	0.14	0.068	**0.149**	96	0.76	[0.50, 1.14]	0.183	**0.386**	0.14	0.128	**0.281**
	CpG_2.3	96	0.63	[0.43, 0.91]	0.013	**0.041**	0.22	0.004	**0.018**	96	0.68	[0.43, 1.06]	0.090	**0.214**	0.27	0.007	**0.030**
	CpG_6	96	0.64	[0.42, 0.96]	0.031	**0.081**	0.27	0.036	**0.092**	96	0.77	[0.47, 1.26]	0.301	**0.561**	0.25	0.374	**0.596**
	CpG_7	96	0.64	[0.42, 0.96]	0.031	**0.081**	0.27	0.036	**0.092**	96	0.77	[0.47, 1.26]	0.301	**0.561**	0.25	0.374	**0.596**
	CpG_8	98	0.97	[0.85, 1.10]	0.645	**0.765**	0.14	0.979	**0.979**	98	1.03	[0.90, 1.18]	0.656	**0.756**	0.22	0.454	**0.670**
	mean	98	0.69	[0.48, 0.98]	0.038	**0.093**	0.18	0.042	**0.103**	98	0.82	[0.54, 1.26]	0.375	**0.616**	0.13	0.272	**0.464**
miR-96, -182, -183	CpG_1	105	0.54	[0.35, 0.84]	0.007	**0.023**	0.16	0.008	**0.027**	105	0.55	[0.32, 0.96]	0.035	**0.099**	0.15	0.009	**0.038**
	CpG_2	105	0.78	[0.53, 1.16]	0.217	**0.369**	0.15	0.302	**0.474**	105	0.80	[0.49, 1.31]	0.386	**0.622**	0.11	0.422	**0.638**
	CpG_3	105	0.50	[0.32, 0.79]	0.003	**0.012**	0.18	0.010	**0.032**	105	0.39	[0.22, 0.72]	0.002	**0.009**	0.13	0.002	**0.012**
	CpG_4.5.6.7.8	105	0.59	[0.39, 0.91]	0.018	**0.053**	0.09	0.004	**0.018**	105	0.60	[0.35, 1.04]	0.068	**0.179**	0.07	0.027	**0.079**
	mean	105	0.54	[0.34, 0.85]	0.008	**0.028**	0.09	0.001	**0.007**	105	0.50	[0.27, 0.91]	0.024	**0.074**	0.12	0.005	**0.023**
miR-96, -182, -183 (b)	CpG_1.2	98	0.87	[0.68, 1.13]	0.308	**0.454**	0.56	0.767	**0.885**	98	0.92	[0.66, 1.30]	0.646	**0.752**	0.65	0.951	**0.959**
	CpG_4	98	0.87	[0.62, 1.22]	0.420	**0.571**	0.84	0.787	**0.899**	98	0.96	[0.62, 1.49]	0.850	**0.898**	0.53	0.979	**0.979**
	CpG_5.6	98	0.73	[0.53, 1.00]	0.050	**0.119**	0.58	0.260	**0.439**	98	0.70	[0.47, 1.04]	0.074	**0.188**	0.58	0.251	**0.454**
	CpG_7.8	98	1.06	[0.74, 1.50]	0.764	**0.861**	0.94	0.858	**0.916**	98	1.16	[0.73, 1.83]	0.534	**0.702**	0.76	0.748	**0.895**
	CpG_9	95	0.82	[0.61, 1.11]	0.196	**0.344**	0.77	0.282	**0.454**	95	0.86	[0.59, 1.26]	0.440	**0.673**	0.58	0.881	**0.936**
	CpG_10.11	98	0.91	[0.72, 1.15]	0.431	**0.580**	0.66	0.461	**0.641**	98	0.92	[0.68, 1.23]	0.562	**0.702**	0.95	0.828	**0.936**
	mean	98	0.85	[0.63, 1.15]	0.297	**0.450**	0.58	0.824	**0.915**	98	0.88	[0.59, 1.29]	0.506	**0.702**	0.87	0.511	**0.727**
miR-129-1	CpG_1	104	0.89	[0.79, 1.01]	0.081	**0.175**	0.31	0.001	**0.006**	104	0.95	[0.79, 1.13]	0.541	**0.702**	0.31	0.011	**0.038**
	CpG_6	104	0.89	[0.79, 1.01]	0.081	**0.175**	0.31	0.001	**0.006**	104	0.95	[0.79, 1.13]	0.541	**0.702**	0.31	0.011	**0.038**
	mean	104	0.89	[0.79, 1.01]	0.081	**0.175**	0.31	0.001	**0.006**	104	0.95	[0.79, 1.13]	0.541	**0.702**	0.31	0.011	**0.038**
miR-129-1 (b)	CpG_1	100	0.73	[0.48, 1.11]	0.146	**0.268**	0.35	0.145	**0.270**	100	0.50	[0.30, 0.84]	0.009	**0.031**	0.35	0.026	**0.076**
	CpG_2	100	0.57	[0.43, 0.77]	0.000	**0.001**	0.62	0.003	**0.018**	100	0.49	[0.34, 0.70]	0.000	**0.001**	0.50	0.000	**0.005**
	CpG_3	75	0.82	[0.64, 1.05]	0.117	**0.232**	0.60	0.174	**0.314**	75	0.71	[0.55, 0.92]	0.009	**0.031**	0.60	0.033	**0.093**
	mean	100	0.68	[0.50, 0.91]	0.010	**0.032**	0.52	0.015	**0.042**	100	0.57	[0.40, 0.80]	0.001	**0.006**	0.39	0.001	**0.009**
miR-132, -212	CpG_18.19	84	1.12	[0.95, 1.33]	0.191	**0.340**	0.06	0.399	**0.582**	84	1.11	[0.87, 1.41]	0.410	**0.635**	0.05	0.263	**0.454**
	CpG_20	80	1.00	[0.62, 1.62]	0.991	**0.991**	0.05	0.961	**0.975**	80	0.84	[0.44, 1.59]	0.588	**0.718**	0.00	0.898	**0.936**
	CpG_21	84	0.98	[0.96, 1.01]	0.268	**0.421**	0.04	0.567	**0.738**	84	0.98	[0.93, 1.03]	0.372	**0.616**	0.04	0.902	**0.936**
	CpG_22	80	1.00	[0.62, 1.62]	0.991	**0.991**	0.05	0.961	**0.975**	80	0.84	[0.44, 1.59]	0.588	**0.718**	0.00	0.898	**0.936**
	mean	85	0.89	[0.62, 1.29]	0.550	**0.694**	0.00	0.192	**0.341**	85	0.75	[0.44, 1.28]	0.285	**0.556**	0.00	0.109	**0.249**
miR-155	CpG_1.2.3	52	0.50	[0.37, 0.67]	<0.000	**<0.000**	0.80	0.005	**0.018**	52	0.52	[0.35, 0.77]	0.001	**0.005**	0.58	0.021	**0.065**
	CpG_4.5	52	0.45	[0.32, 0.64]	<0.000	**<0.000**	0.81	0.003	**0.018**	52	0.50	[0.32, 0.79]	0.003	**0.011**	0.14	0.056	**0.145**
	CpG_8.9.10	52	0.42	[0.27, 0.67]	0.000	**0.001**	0.50	0.002	**0.010**	52	0.41	[0.23, 0.73]	0.002	**0.009**	0.50	0.005	**0.023**
	mean	52	0.36	[0.23, 0.56]	<0.000	**<0.000**	0.67	0.005	**0.018**	52	0.39	[0.22, 0.68]	0.001	**0.005**	0.60	0.019	**0.062**
miR-155 (b)	CpG_1.2.3	72	0.42	[0.25, 0.69]	0.001	**0.003**	0.08	0.004	**0.018**	72	0.27	[0.15, 0.51]	<0.000	**0.001**	0.10	0.000	**0.004**
	CpG_4	73	0.45	[0.30, 0.67]	<0.000	**0.001**	0.24	0.005	**0.018**	73	0.42	[0.26, 0.67]	0.000	**0.002**	0.53	0.004	**0.020**
	CpG_5.6	72	0.33	[0.18, 0.62]	0.001	**0.002**	0.29	0.002	**0.011**	72	0.21	[0.09, 0.47]	0.000	**0.001**	0.29	0.000	**0.004**
	CpG_7	73	0.51	[0.36, 0.73]	0.000	**0.001**	0.63	0.011	**0.033**	73	0.41	[0.26, 0.63]	<0.000	**0.001**	0.58	0.003	**0.016**
	mean	73	0.47	[0.32, 0.68]	<0.000	**0.001**	0.55	0.004	**0.018**	73	0.36	[0.22, 0.58]	<0.000	**0.001**	0.55	0.000	**0.004**
miR-195, -497	CpG_1	87	0.95	[0.78, 1.15]	0.592	**0.712**	0.00	0.131	**0.260**	87	1.03	[0.82, 1.29]	0.798	**0.854**	0.00	0.371	**0.596**
	CpG_3	87	0.93	[0.74, 1.17]	0.562	**0.694**	0.00	0.136	**0.265**	87	1.04	[0.81, 1.35]	0.757	**0.842**	0.00	0.382	**0.600**
	CpG_4	87	0.97	[0.81, 1.17]	0.783	**0.861**	0.52	0.840	**0.916**	87	1.01	[0.81, 1.26]	0.946	**0.954**	0.21	0.523	**0.727**
	CpG_5	87	0.75	[0.60, 0.93]	0.010	**0.032**	0.51	0.014	**0.042**	87	0.61	[0.40, 0.92]	0.020	**0.064**	0.29	0.017	**0.056**
	CpG_6.7	87	0.65	[0.45, 0.93]	0.018	**0.053**	0.29	0.010	**0.032**	87	0.56	[0.33, 0.94]	0.027	**0.079**	0.29	0.037	**0.102**
	CpG_8	87	1.02	[0.81, 1.29]	0.866	**0.919**	0.02	0.865	**0.916**	87	1.04	[0.79, 1.37]	0.794	**0.854**	0.02	0.856	**0.936**
	CpG_9	87	1.03	[0.92, 1.16]	0.559	**0.694**	0.11	0.680	**0.840**	87	0.95	[0.77, 1.16]	0.606	**0.724**	0.00	0.905	**0.936**
	CpG_10	87	1.03	[0.97, 1.08]	0.336	**0.478**	0.05	0.564	**0.738**	87	0.93	[0.80, 1.08]	0.329	**0.594**	0.00	0.586	**0.780**
	CpG_11.12.13	85	1.14	[0.91, 1.43]	0.263	**0.421**	0.05	0.651	**0.829**	85	1.09	[0.83, 1.43]	0.531	**0.702**	0.05	0.745	**0.895**
	mean	87	0.80	[0.61, 1.06]	0.117	**0.232**	0.17	0.286	**0.455**	87	0.72	[0.48, 1.08]	0.109	**0.249**	0.17	0.392	**0.608**

continued at part b

Supplementary Table 1b

continued from part a

NOA-04		Progression Free Survival								Overall Survival							
		Cox regression					Kaplan-Meier estimate			Cox regression					Kaplan-Meier estimate		
		Obs	HazR	95% CI	p-value	**p-value adj**	cut	cut p-value	**cut p-value adj**	Obs	HazR	95% CI	p-value	**p-value adj**	cut	cut p-value	**cut p-value adj**
**miR-200a+b, -429**	CpG_1	98	0.91	[0.67, 1.24]	0.555	**0.694**	0.49	0.660	**0.831**	98	0.90	[0.61, 1.35]	0.616	**0.724**	0.52	0.520	**0.727**
	CpG_2	98	0.89	[0.74, 1.07]	0.227	**0.382**	0.94	0.350	**0.526**	98	0.93	[0.73, 1.19]	0.550	**0.702**	0.97	0.809	**0.924**
	CpG_3	97	0.97	[0.83, 1.13]	0.661	**0.769**	0.97	0.967	**0.975**	97	1.08	[0.84, 1.40]	0.546	**0.702**	0.96	0.754	**0.895**
	CpG_4.5	98	0.88	[0.66, 1.19]	0.415	**0.571**	0.96	0.324	**0.502**	98	0.95	[0.64, 1.41]	0.789	**0.854**	0.96	0.167	**0.355**
	CpG_7	92	0.85	[0.72, 1.00]	0.044	**0.106**	0.79	0.046	**0.109**	92	0.86	[0.70, 1.06]	0.154	**0.334**	0.79	0.112	**0.252**
	CpG_8	98	1.01	[0.96, 1.06]	0.758	**0.861**	0.99	0.760	**0.885**	98	1.02	[0.95, 1.09]	0.613	**0.724**	0.99	0.777	**0.912**
	mean	98	0.85	[0.66, 1.09]	0.201	**0.347**	0.90	0.417	**0.596**	98	0.91	[0.65, 1.26]	0.560	**0.702**	0.90	0.721	**0.895**
**miR-210**	CpG_1	76	0.27	[0.15, 0.49]	<0.000	**0.000**	0.30	0.000	**0.004**	76	0.27	[0.13, 0.56]	0.000	**0.003**	0.30	0.002	**0.014**
	CpG_2	73	0.27	[0.15, 0.47]	<0.000	**<0.000**	0.30	0.000	**0.004**	73	0.25	[0.12, 0.52]	0.000	**0.001**	0.17	0.001	**0.009**
	CpG_5	74	0.58	[0.44, 0.78]	0.000	**0.001**	0.98	0.003	**0.015**	74	0.71	[0.48, 1.03]	0.070	**0.180**	0.98	0.058	**0.145**
	CpG_6	70	0.29	[0.17, 0.52]	<0.000	**0.000**	0.46	0.000	**0.004**	70	0.28	[0.14, 0.58]	0.001	**0.004**	0.46	0.011	**0.038**
	CpG_7	76	0.25	[0.14, 0.46]	<0.000	**<0.000**	0.57	0.000	**0.003**	76	0.19	[0.09, 0.42]	<0.000	**0.001**	0.32	0.000	**0.005**
	CpG_8	76	0.23	[0.12, 0.44]	<0.000	**<0.000**	0.46	0.000	**0.002**	76	0.18	[0.08, 0.40]	<0.000	**0.001**	0.46	0.000	**0.004**
	CpG_9	76	0.19	[0.11, 0.35]	<0.000	**<0.000**	0.33	0.000	**0.002**	76	0.13	[0.06, 0.28]	<0.000	**<0.000**	0.33	0.000	**<0.000**
	CpG_10	76	0.85	[0.58, 1.24]	0.392	**0.551**	0.06	0.015	**0.042**	76	0.97	[0.62, 1.53]	0.910	**0.929**	0.07	0.044	**0.115**
	CpG_12	76	0.35	[0.21, 0.61]	0.000	**0.001**	0.42	0.001	**0.006**	76	0.28	[0.14, 0.54]	0.000	**0.001**	0.42	0.000	**0.005**
	mean	76	0.23	[0.13, 0.41]	<0.000	**<0.000**	0.70	0.000	**0.002**	76	0.21	[0.10, 0.43]	<0.000	**0.001**	0.43	0.001	**0.005**
**miR-335**	CpG_1.2	97	0.58	[0.44, 0.77]	0.000	**0.001**	0.39	0.004	**0.018**	97	0.57	[0.40, 0.79]	0.001	**0.004**	0.38	0.005	**0.023**
	CpG_3	97	0.66	[0.45, 0.97]	0.035	**0.088**	0.58	0.062	**0.142**	97	0.53	[0.33, 0.85]	0.008	**0.029**	0.58	0.011	**0.038**
	CpG_4	97	0.57	[0.41, 0.79]	0.001	**0.004**	0.44	0.010	**0.032**	97	0.48	[0.33, 0.72]	0.000	**0.002**	0.32	0.002	**0.012**
	CpG_5	96	0.57	[0.42, 0.76]	0.000	**0.001**	0.61	0.004	**0.018**	96	0.54	[0.38, 0.77]	0.001	**0.004**	0.61	0.005	**0.023**
	mean	97	0.55	[0.40, 0.77]	0.001	**0.002**	0.43	0.006	**0.022**	97	0.50	[0.33, 0.74]	0.001	**0.004**	0.40	0.004	**0.020**
**miR-335 (b)**	CpG_1	83	0.78	[0.49, 1.25]	0.305	**0.454**	0.04	0.261	**0.439**	83	1.05	[0.57, 1.93]	0.880	**0.910**	0.28	0.904	**0.936**
	CpG_2.3	85	1.07	[0.71, 1.61]	0.735	**0.846**	0.15	0.763	**0.885**	85	1.23	[0.72, 2.11]	0.450	**0.673**	0.23	0.731	**0.895**
	CpG_4	85	1.03	[0.69, 1.55]	0.880	**0.926**	0.14	0.965	**0.975**	85	1.05	[0.62, 1.78]	0.861	**0.898**	0.53	0.798	**0.919**
	CpG_5.6	86	1.15	[0.75, 1.78]	0.513	**0.661**	0.96	0.851	**0.916**	86	1.29	[0.74, 2.25]	0.373	**0.616**	0.31	0.371	**0.596**
	CpG_7	83	1.16	[0.79, 1.71]	0.448	**0.596**	0.71	0.483	**0.658**	83	1.19	[0.73, 1.94]	0.497	**0.702**	0.71	0.741	**0.895**
	mean	86	1.06	[0.68, 1.67]	0.793	**0.864**	0.67	0.823	**0.915**	86	1.22	[0.68, 2.20]	0.499	**0.702**	0.91	0.546	**0.743**
**miR-1305**	CpG_1.2.3	51	0.62	[0.34, 1.16]	0.133	**0.248**	0.21	0.352	**0.526**	51	0.46	[0.20, 1.04]	0.061	**0.164**	0.18	0.188	**0.378**
	CpG_5.6	51	0.63	[0.34, 1.15]	0.133	**0.248**	0.24	0.065	**0.147**	51	0.69	[0.34, 1.42]	0.311	**0.571**	0.24	0.521	**0.727**
	CpG_7.8	50	0.86	[0.60, 1.24]	0.420	**0.571**	0.05	0.674	**0.840**	50	0.80	[0.49, 1.31]	0.375	**0.616**	0.04	0.631	**0.830**
	CpG_9	51	0.88	[0.75, 1.03]	0.111	**0.227**	0.32	0.140	**0.265**	51	0.83	[0.65, 1.07]	0.144	**0.316**	0.32	0.257	**0.454**
	CpG_10	51	0.73	[0.46, 1.16]	0.179	**0.323**	0.16	0.140	**0.265**	51	0.85	[0.50, 1.45]	0.559	**0.702**	0.02	0.256	**0.454**
	CpG_11.12	51	0.59	[0.32, 1.09]	0.091	**0.193**	0.09	0.160	**0.293**	51	0.73	[0.35, 1.52]	0.406	**0.635**	0.07	0.198	**0.392**
	CpG_13	50	0.98	[0.80, 1.19]	0.811	**0.876**	0.00	0.434	**0.610**	50	1.06	[0.87, 1.29]	0.594	**0.718**	0.18	0.470	**0.686**
	mean	51	0.56	[0.32, 0.95]	0.033	**0.084**	0.33	0.261	**0.439**	51	0.53	[0.26, 1.08]	0.079	**0.192**	0.10	0.240	**0.447**

continued at part c

Supplementary Table 1c

continued from part b

GGN		Progression Free Survival								Overall Survival							
		Cox regression					Kaplan-Meier estimate			Cox regression					Kaplan-Meier estimate		
		Obs	HazR	95% CI	p-value	**p-value adj**	cut	cut p-value	**cut p-value adj**	Obs	HazR	95% CI	p-value	**p-value adj**	cut	cut p-value	**cut p-value adj**
**miR-10b**	CpG_1	79	1.17	[0.74, 1.85]	0.512	**0.576**	0.53	0.523	**0.565**	79	1.37	[0.70, 2.67]	0.356	**0.447**	0.24	0.416	**0.432**
	CpG_2.3.4	79	1.65	[1.04, 2.62]	0.033	**0.071**	0.48	0.007	**0.050**	79	1.79	[0.92, 3.48]	0.088	**0.135**	0.36	0.149	**0.187**
	CpG_5	73	1.97	[1.10, 3.54]	0.023	**0.060**	0.35	0.035	**0.090**	73	1.99	[0.87, 4.54]	0.102	**0.149**	0.54	0.155	**0.190**
	CpG_6	79	1.43	[0.95, 2.16]	0.085	**0.123**	0.21	0.044	**0.098**	79	1.84	[1.07, 3.16]	0.028	**0.050**	0.21	0.012	**0.024**
	mean	79	1.51	[1.00, 2.26]	0.049	**0.089**	0.38	0.037	**0.090**	79	1.70	[0.96, 3.00]	0.066	**0.105**	0.38	0.053	**0.072**
**miR-34bc**	CpG_1	81	0.61	[0.39, 0.95]	0.028	**0.063**	0.32	0.049	**0.102**	81	0.54	[0.28, 1.02]	0.057	**0.093**	0.19	0.049	**0.071**
	CpG_2.3	81	0.76	[0.50, 1.17]	0.218	**0.306**	0.30	0.191	**0.252**	81	0.74	[0.40, 1.38]	0.342	**0.444**	0.30	0.194	**0.232**
	CpG_6	81	1.06	[0.69, 1.63]	0.782	**0.812**	0.12	0.645	**0.657**	81	1.05	[0.57, 1.92]	0.881	**0.881**	0.10	0.280	**0.304**
	CpG_7	81	1.06	[0.69, 1.63]	0.782	**0.812**	0.12	0.645	**0.657**	81	1.05	[0.57, 1.92]	0.881	**0.881**	0.10	0.280	**0.304**
	CpG_8	81	1.06	[0.90, 1.24]	0.506	**0.576**	0.06	0.287	**0.330**	81	1.11	[0.90, 1.36]	0.346	**0.444**	0.04	0.413	**0.432**
	mean	81	0.86	[0.57, 1.30]	0.475	**0.576**	0.16	0.063	**0.113**	81	0.84	[0.47, 1.53]	0.574	**0.639**	0.12	0.054	**0.072**
**miR-96, -182, -183**	CpG_1	80	0.76	[0.48, 1.19]	0.228	**0.306**	0.17	0.163	**0.220**	80	0.85	[0.47, 1.55]	0.605	**0.654**	0.11	0.428	**0.436**
	CpG_2	80	0.88	[0.62, 1.25]	0.476	**0.576**	0.72	0.743	**0.743**	80	0.87	[0.54, 1.41]	0.579	**0.639**	0.43	0.712	**0.712**
	CpG_3	80	0.61	[0.36, 1.04]	0.072	**0.111**	0.19	0.033	**0.090**	80	0.62	[0.30, 1.31]	0.215	**0.306**	0.14	0.011	**0.023**
	CpG_4.5.6.7.8	80	0.81	[0.51, 1.26]	0.348	**0.448**	0.08	0.225	**0.288**	80	0.76	[0.41, 1.41]	0.381	**0.468**	0.08	0.143	**0.183**
	mean	80	0.76	[0.49, 1.19]	0.225	**0.306**	0.12	0.231	**0.288**	80	0.78	[0.42, 1.42]	0.415	**0.497**	0.12	0.016	**0.029**
**miR-96, -182, -183 (b)**	CpG_1.2	82	0.95	[0.77, 1.17]	0.609	**0.658**	0.77	0.242	**0.291**	82	0.90	[0.67, 1.20]	0.463	**0.544**	0.67	0.047	**0.070**
	CpG_4	82	0.94	[0.76, 1.17]	0.597	**0.657**	0.75	0.259	**0.304**	82	0.91	[0.66, 1.26]	0.580	**0.639**	0.72	0.259	**0.297**
	CpG_5.6	82	0.79	[0.61, 1.01]	0.065	**0.102**	0.71	0.053	**0.102**	82	0.65	[0.47, 0.90]	0.010	**0.022**	0.61	0.008	**0.019**
	CpG_7.8	82	0.99	[0.79, 1.25]	0.951	**0.951**	0.71	0.587	**0.622**	82	0.95	[0.68, 1.33]	0.775	**0.805**	0.69	0.281	**0.304**
	CpG_9	82	0.93	[0.76, 1.14]	0.498	**0.576**	0.68	0.235	**0.288**	82	0.86	[0.65, 1.13]	0.267	**0.370**	0.68	0.031	**0.049**
	CpG_10.11	82	0.98	[0.82, 1.17]	0.809	**0.824**	0.78	0.372	**0.409**	82	0.94	[0.73, 1.22]	0.652	**0.691**	0.76	0.050	**0.071**
	mean	82	0.93	[0.75, 1.15]	0.488	**0.576**	0.74	0.295	**0.332**	82	0.86	[0.64, 1.15]	0.302	**0.408**	0.63	0.056	**0.073**
**miR-129-1**	CpG_1	50	0.46	[0.23, 0.91]	0.026	**0.062**	0.37	0.014	**0.065**	50	0.45	[0.17, 1.16]	0.096	**0.145**	0.44	0.223	**0.261**
	CpG_2	78	0.68	[0.47, 0.97]	0.036	**0.072**	0.52	0.115	**0.163**	78	0.48	[0.29, 0.79]	0.004	**0.010**	0.55	0.008	**0.019**
	mean	78	0.67	[0.46, 0.99]	0.047	**0.089**	0.48	0.101	**0.151**	78	0.48	[0.28, 0.83]	0.008	**0.018**	0.51	0.006	**0.017**
**miR-155**	CpG_1.2.3	80	0.83	[0.73, 0.95]	0.006	**0.026**	0.23	0.073	**0.120**	80	0.77	[0.66, 0.90]	0.001	**0.004**	0.23	0.005	**0.015**
	CpG_4.5	80	0.86	[0.77, 0.96]	0.007	**0.026**	0.22	0.074	**0.120**	80	0.80	[0.70, 0.91]	0.001	**0.004**	0.22	0.005	**0.015**
	CpG_8.9.10	80	0.81	[0.66, 1.00]	0.051	**0.089**	0.19	0.046	**0.099**	80	0.65	[0.50, 0.83]	0.001	**0.004**	0.19	0.001	**0.007**
	mean	80	0.82	[0.70, 0.96]	0.012	**0.037**	0.21	0.092	**0.142**	80	0.72	[0.60, 0.87]	0.001	**0.004**	0.21	0.006	**0.017**
**miR-155 (b)**	CpG_1.2.3	80	0.75	[0.59, 0.97]	0.026	**0.062**	0.50	0.051	**0.102**	80	0.64	[0.46, 0.88]	0.007	**0.016**	0.09	0.012	**0.024**
	CpG_4	80	0.73	[0.57, 0.93]	0.011	**0.036**	0.14	0.070	**0.120**	80	0.61	[0.44, 0.83]	0.002	**0.006**	0.14	0.004	**0.014**
	CpG_5.6	80	0.63	[0.44, 0.91]	0.012	**0.037**	0.11	0.040	**0.093**	80	0.53	[0.32, 0.88]	0.015	**0.030**	0.05	0.002	**0.009**
	CpG_7	80	0.73	[0.60, 0.90]	0.003	**0.025**	0.76	0.014	**0.065**	80	0.63	[0.48, 0.83]	0.001	**0.004**	0.10	0.003	**0.014**
	mean	80	0.70	[0.54, 0.91]	0.007	**0.026**	0.65	0.023	**0.072**	80	0.59	[0.42, 0.83]	0.002	**0.007**	0.10	0.004	**0.015**
**miR-210**	CpG_1	80	0.48	[0.29, 0.79]	0.004	**0.025**	0.30	0.001	**0.050**	80	0.31	[0.16, 0.60]	0.001	**0.004**	0.30	0.001	**0.005**
	CpG_2	80	0.51	[0.32, 0.82]	0.005	**0.026**	0.40	0.003	**0.050**	80	0.33	[0.17, 0.63]	0.001	**0.004**	0.40	0.000	**0.004**
	CpG_5	80	0.60	[0.45, 0.80]	0.000	**0.022**	0.66	0.007	**0.050**	80	0.52	[0.37, 0.73]	0.000	**0.002**	0.66	0.000	**0.004**
	CpG_6	79	0.49	[0.30, 0.81]	0.005	**0.026**	0.27	0.017	**0.066**	79	0.29	[0.14, 0.59]	0.001	**0.004**	0.41	0.001	**0.007**
	CpG_7	80	0.46	[0.29, 0.74]	0.001	**0.022**	0.20	0.004	**0.050**	80	0.28	[0.15, 0.52]	<0.000	**0.002**	0.57	0.000	**0.004**
	CpG_8	80	0.46	[0.28, 0.77]	0.003	**0.025**	0.18	0.007	**0.050**	80	0.25	[0.12, 0.51]	0.000	**0.002**	0.54	0.000	**0.004**
	CpG_9	80	0.62	[0.41, 0.92]	0.018	**0.050**	0.36	0.013	**0.065**	80	0.35	[0.20, 0.60]	0.000	**0.002**	0.67	0.000	**0.004**
	CpG_10	80	0.61	[0.38, 0.97]	0.036	**0.072**	0.23	0.017	**0.066**	80	0.44	[0.21, 0.88]	0.021	**0.039**	0.22	0.013	**0.024**
	CpG_12	80	0.51	[0.34, 0.78]	0.002	**0.022**	0.16	0.002	**0.050**	80	0.38	[0.21, 0.67]	0.001	**0.004**	0.49	0.007	**0.019**
	mean	80	0.46	[0.28, 0.75]	0.002	**0.022**	0.23	0.008	**0.050**	80	0.27	[0.14, 0.53]	0.000	**0.002**	0.59	0.000	**0.004**
**miR-335**	CpG_1.2	81	0.74	[0.54, 1.00]	0.051	**0.089**	0.63	0.087	**0.139**	81	0.61	[0.41, 0.91]	0.017	**0.032**	0.63	0.031	**0.049**
	CpG_3	81	0.81	[0.58, 1.14]	0.233	**0.306**	0.49	0.103	**0.151**	81	0.61	[0.39, 0.95]	0.029	**0.051**	0.47	0.035	**0.054**
	CpG_4	81	0.75	[0.55, 1.01]	0.061	**0.099**	0.54	0.061	**0.113**	81	0.64	[0.43, 0.96]	0.030	**0.051**	0.56	0.022	**0.038**
	CpG_5	81	0.73	[0.53, 1.01]	0.060	**0.099**	0.61	0.161	**0.220**	81	0.56	[0.37, 0.85]	0.006	**0.015**	0.61	0.017	**0.029**
	mean	81	0.75	[0.54, 1.03]	0.076	**0.114**	0.55	0.024	**0.072**	81	0.60	[0.40, 0.90]	0.015	**0.030**	0.55	0.010	**0.023**

Supplementary Table 2 No correlation of candidate methylation was seen with the survival of elderly patients with malignant astrocytoma of the NOA-08 trial. The NOA-08 trail was composed of patients older than 65 years with mostly glioblastoma (89%) and anaplastic glioma (11%) (Wick et al. 2012). The candidate methylation was correlated with the survival as previously shown for NOA-04 and GGN by Cox regression. In the case of the cluster miR-96, -182, -183 and miR-155 a second region was analyzed marked by (b). Obs: observation, number of patients with full data; HazR: Hazard ratio; CI: confidence interval; p-value adj: p-value adjusted for testing of multiple amplicons

**NOA-08**	**Progression Free Survival**					**Overall Survival**				
	Obs	HazR	95% CI	p-value	**p-value adj**	Obs	HazR	95% CI	p-value	**p-value adj**
**mir-10b**	72	1.07	[0.76, 1.50]	0.718	0.998	72	1.03	[0.77, 1.39]	0.833	0.969
**mir-34bc**	75	0.88	[0.64, 1.22]	0.440	0.998	75	0.88	[0.68, 1.14]	0.323	0.630
**mir-96, -182, -183 (b)**	78	0.96	[0.67, 1.35]	0.800	0.998	78	1.02	[0.73, 1.41]	0.922	0.969
**mir-155**	85	1.17	[0.80, 1.72]	0.412	0.998	85	0.87	[0.60, 1.26]	0.459	0.690
**mir-155 (b)**	60	0.94	[0.60, 1.49]	0.804	0.998	60	0.77	[0.50, 1.16]	0.209	0.533
**mir-210**	91	0.97	[0.80, 1.19]	0.794	0.998	91	0.93	[0.78, 1.12]	0.455	0.690
**mir-335**	101	1.01	[0.74, 1.39]	0.944	0.998	101	0.90	[0.66, 1.22]	0.501	0.690

Supplementary Table 3 No prognostic miRNA methylation calculated in glioblastoma patients. The promoter methylation was correlated with the survival in TCGA patients with glioblastomas. Obs: observations; HazR: Hazard ratio; CI: confidence interval

	**Overall Survival**			
	Obs	HazR	95 % CI	**p-value**
**miR-155**	104	1.08	[0.97, 1.18]	**0.133**
**miR-210**	104	1.16	[0.65, 2.04]	**0.418**
**miR-335**	104	0.94	[0.81, 1.09]	**0.410**

Supplementary Table 4 Multivariate Cox regression analysis including molecular classification on NOA-04 and TCGA patients. Multivariate Cox regression models were performed to test the prognostic relevance of the miRNA methylation taken into account the new molecular classification [4]. Thus, IDH status was subdivided according to the molecular classification (Mol) into CpG island methylator phenotype (CIMP) negative vs CIMP positive 1p/19q non-codeleted and CIMP negative vs CIMP positive with 1p/19q codeleted. The results for the NOA-04 and TCGA patients using the established factors are found in the main article (Figure 4). Significant correlations were denoted in grey (p-value < 0.05). HazR: Hazard ratio; CI: confidence interval; CIMP: CpG island methylator phenotype; C^-: CIMP negative; $C^{+/-}$: CIMP positive, non-codeleted; $C^{+/+}$: CIMP positive, 1p/19q codeleted; m: methylated; u: unmethylated; O(A): oligodendroglioma and oligoastrocytoma; A: astrocytoma; RT: radiotherapy; CT: chemotherapy

NOA-04 - Progression Free Survival

	mir-155			mir-210			mir-335		
	HazR	95 % CI	p-value	HazR	95 % CI	p-value	HazR	95 % CI	p-value
**miRNA meth.**	0.14	[0.02, 0.82]	**0.029**	0.33	[0.04, 2.29]	**0.260**	1.37	[0.36, 5.24]	**0.640**
Mol $C^{+/-}/C^-$	0.56	[0.19, 1.68]	**0.300**	0.35	[0.13, 0.89]	**0.029**	0.30	[0.13, 0.67]	**0.003**
Mol $C^{+/+}/C^-$	0.43	[0.14, 1.36]	**0.150**	0.27	[0.06, 1.11]	**0.070**	0.11	[0.04, 0.31]	**0.000**
MGMT m/u	0.66	[0.27, 1.65]	**0.375**	0.81	[0.39, 1.67]	**0.570**	0.74	[0.42, 1.3]	**0.300**
Histology O(A)/A	0.79	[0.31, 2.03]	**0.630**	0.68	[0.35, 1.306]	**0.250**	0.78	[0.43, 1.4]	**0.400**
Therapy RT/CT	0.79	[0.33, 1.93]	**0.610**	1.32	[0.68, 2.52]	**0.410**	1.28	[0.74, 2.23]	**0.370**

TCGA - Overall Survival

	mir-155			mir-210			mir-335		
	HazR	95 % CI	p-value	HazR	95 % CI	p-value	HazR	95 % CI	p-value
**miRNA meth.**	0.04	[0.01, 0.22]	**0.000**	0.12	[0.00, 6.21]	**0.288**	0.98	[0.05, 21.1]	**0.990**
Mol $C^{+/-}/C^-$	0.63	[0.16, 2.57]	**0.520**	0.21	[0.06, 0.74]	**0.016**	0.16	[0.05, 0.55]	**0.003**
Mol $C^{+/+}/C^-$	0.50	[0.11, 2.32]	**0.390**	0.25	[0.05, 1.26]	**0.094**	0.16	[0.04, 0.70]	**0.015**
MGMT m/u	1.34	[0.47, 3.81]	**0.580**	2.07	[0.68, 6.27]	**0.190**	1.95	[0.62, 6.07]	**0.247**
Histology O(A)/A	0.68	[0.28, 1.64]	**0.400**	0.91	[0.38, 2.15]	**0.830**	0.90	[0.39, 2.12]	**0.817**

Supplementary Table 5 Uni- and multivariate Cox regression analysis on progression free survival of GGN patients. Candidate methylation (meth.), *IDH* mutation status, *MGMT* promoter methylation status, histopathology and first line treatment were taken into account for the progression free survival of the GGN patients. Additionally, the univariate Cox regression calculations on the GGN patients are given for *IDH* mutation, *MGMT* methylation and histopathology. Significant correlations were denoted in grey (p-value < 0.05). HazR: Hazard ratio; CI: confidence interval; mut: mutated; wt: wild type; m: methylated; u: unmethylated; O(A): oligodendroglioma and oligoastrocytoma; A: astrocytoma; RT: radiotherapy; CT: chemotherapy; n: number of patients with data for all five factors; events: number of patients with progress/death

GGN - Progression Free Survival

	**miR-155**			**miR-210**			**miR-335**		
	HazR	95 % CI	**p-value**	HazR	95 % CI	**p-value**	HazR	95 % CI	**p-value**
**miRNA meth.**	0.61	[0.09, 4.34]	**0.620**	0.08	[0.03, 0.93]	**0.060**	0.31	[0.03, 2.92]	**0.300**
IDH mut/wt	0.56	[0.22, 1.41]	**0.220**	0.77	[0.91, 4.90]	**0.570**	0.59	[0.25, 1.42]	**0.240**
MGMT m/u	1.40	[0.47, 4.13]	**0.540**	2.19	[0.59, 2.59]	**0.160**	1.51	[0.56, 4.08]	**0.420**
Histology O(A)/A	0.45	[0.20, 0.98]	**0.040**	0.61	[0.36, 1.21]	**0.240**	0.42	[0.20, 0.88]	**0.020**
Therapy RT/CT	1.12	[0.55, 2.29]	**0.750**	1.16	[0.67, 2.62]	**0.690**	1.18	[0.56, 2.49]	**0.670**
	n = 66, events = 37			n = 66, events = 36			n = 67, events = 36		

Progression Free Survival

	HazR	95% CI	**p-value adj**
IDH mut/wt	0.57	[0.31, 1.06]	**0.070**

Progression Free Survival

	HazR	95% CI	**p-value adj**
MGMT m/u	0.79	[0.36, 1.73]	**0.600**

Progression Free Survival

	HazR	95% CI	**p-value adj**
Histology O(A)/A	0.34	[0.18, 0.65]	**0.001**
